# Connectivity of Triangulation Flip Graphs in the Plane

**DOI:** 10.1007/s00454-022-00436-2

**Published:** 2022-11-14

**Authors:** Uli Wagner, Emo Welzl

**Affiliations:** 1 IST Austria, Am Campus 1, 3400 Klosterneuburg, Austria; 2 Department of Computer Science, ETH Zürich, 8092 Zürich, Switzerland

**Keywords:** (Regular) Triangulation, (Bistellar) Flip graph, Graph connectivity, Secondary polytope, (Simultaneously) Flippable edges, Menger’s Theorem, 05C10, 05C40, 52B05, 52C35, 52C45, 68U05, 68R10

## Abstract

Given a finite point set *P* in *general position* in the plane, a *full triangulation* of *P* is a maximal straight-line embedded plane graph on *P*. A *partial triangulation* of *P* is a full triangulation of some subset $P'$ of *P* containing all extreme points in *P*. A *bistellar flip* on a partial triangulation either flips an edge (called *edge flip*), removes a non-extreme point of degree 3, or adds a point in $P \setminus P'$ as vertex of degree 3. The *bistellar flip graph* has all partial triangulations as vertices, and a pair of partial triangulations is adjacent if they can be obtained from one another by a bistellar flip. The *edge flip graph* is defined with full triangulations as vertices, and edge flips determining the adjacencies. Lawson showed in the early seventies that these graphs are connected. The goal of this paper is to investigate the structure of these graphs, with emphasis on their vertex connectivity. For sets *P* of *n* points in the plane in general position, we show that the edge flip graph is $\lceil {n}/{2}-2\rceil$-vertex connected, and the bistellar flip graph is $(n-3)$-vertex connected; both results are tight. The latter bound matches the situation for the subfamily of regular triangulations (i.e., partial triangulations obtained by lifting the points to 3-space and projecting back the lower convex hull), where $(n-3)$-vertex connectivity has been known since the late eighties through the secondary polytope due to Gelfand, Kapranov, & Zelevinsky and Balinski’s Theorem. For the edge flip-graph, we additionally show that the vertex connectivity is at least as large as (and hence equal to) the minimum degree (i.e., the minimum number of flippable edges in any full triangulation), provided that *n* is large enough. Our methods also yield several other results: (i) The edge flip graph can be covered by graphs of polytopes of dimension $\lceil {n}/{2} -2\rceil$ (products of associahedra) and the bistellar flip graph can be covered by graphs of polytopes of dimension $n-3$ (products of secondary polytopes). (ii) A partial triangulation is regular, if it has distance $n-3$ in the Hasse diagram of the partial order of partial subdivisions from the trivial subdivision. (iii) All partial triangulations of a point set are regular iff the partial order of partial subdivisions has height $n-3$. (iv) There are arbitrarily large sets *P* with non-regular partial triangulations and such that every proper subset has only regular triangulations, i.e., there are no small certificates for the existence of non-regular triangulations.

## Introduction

Triangulations of point sets play a role in many areas including mathematics, numerics, computer science, and processing of geographic data, [4, 11, 22, 24]. A natural way to provide structure to the *set of all triangulations* is to consider a graph, called *flip graph*, with the triangulations as vertices and with pairs of triangulations adjacent if they can be obtained from each other by a minimal local change, called a *flip* (see below for the precise definition). One of the first and most prominent results on flip graphs of planar points sets (for edge flips), proved by Lawson [21] in 1972, is that they are connected. The corresponding question of connectedness of flip graphs in higher dimensions remained a mystery until Santos [33] showed, in 2000, that in dimension $5$ and higher, there exist point sets for which the graph (for bistellar flips) is not connected. The question is still open in dimension $3$ and $4$.

Here, we concentrate on point sets in general position in the plane and investigate “how” connected the flip graphs are, i.e., determine the largest *k* (in terms of *n*, the size of the underlying point set) such that *k*-vertex connectivity holds. Moreover, we supply some structural results for the flip graph.

The preceding discussion swept under the rug that there are several types of triangulations, most prominently *full triangulations* (as mostly considered in computational geometry), *partial triangulations* (as primarily considered in discrete geometry), and *regular triangulations* (also called coherent triangulations or weighted Delaunay triangulations). We will address the vertex connectivity for full and partial triangulations, and we will discuss some implications for regular triangulations, for which the vertex connectivity of the flip graph has been known since the late eighties via the secondary polytope introduced by Gelfand et al. [15] (see [24, Corollary 5.3.2]).

Let us first supply the basic definitions and then discuss the results in more detail.

### Notation

We let $\mathbb {N}$ denote the set of positive integers and we let $\mathbb {N}_0:= \mathbb {N}\cup \{0\}$. We write $A \oplus B$ for the symmetric difference of sets *A* and *B*.

### Definition 1.1

(*point set*). Throughout this paper we let *P* denote a finite planar point set in *general position* (i.e., no three points on a line) with $n\ge 3$ points. The set of *extreme points* of *P* (i.e., the vertices of the convex hull of *P*) is denoted by ${{\,\mathrm{\textsf {xtr}\mathrm }\,}}{P}$, and we let $P^\circ := P \setminus {{\,\mathrm{\textsf {xtr}\mathrm }\,}}{P}$ denote the set of *inner* (i.e., non-extreme) *points* in *P*. We consistently use the notation $h=h(P):= |{{{\,\mathrm{\textsf {xtr}\mathrm }\,}}{P}}|$ and $n^{\circ }=n^{\circ }(P):=|P^\circ |=n-h$, and we let ${\textsf {E} _\textsf {hull} }= {\textsf {E} _\textsf {hull} }(P) \subseteq {P \atopwithdelims ()2}$ denote the set of edges of the convex hull of *P*, $|{\textsf {E} _\textsf {hull} }|=h$.

### Definition 1.2

(*plane graph*). For graphs $G=(P',E)$, $P' \subseteq P$, $E \subseteq {P' \atopwithdelims ()2}$, on $P'$ we often identify edges $\{p,q\}\in E$ with their corresponding straight line segments *pq*. We let ${\textsf {V} }G := P'$ and $\textsf {E} G:=E$. A graph *G* on *P* is *plane* if no two straight line segments corresponding to edges in $\textsf {E} G$ cross (i.e., they are disjoint except for possibly sharing an endpoint). For *G* plane, the bounded connected components of the complement of the union of the edges are called *regions*, the set of regions of *G* is denoted by ${\textsf {R} }G$.

Note that regions are *open sets*. Note also that isolated points in a plane graph are ignored in the definition of regions.

### Definition 1.3

(*full, partial, regular triangulation*).

- A *full triangulation* of *P* is a maximal plane graph $T=(P,E)$.
- A *partial triangulation* of *P* is a full triangulation $T=(P',E)$ with ${{\,\mathrm{\textsf {xtr}\mathrm }\,}}{P} \subseteq P' \subseteq P$ (hence ${\textsf {E} _\textsf {hull} }\subseteq \textsf {E} T$).
- A *regular triangulation* of *P* is a triangulation obtained by projecting (back to $\mathbb {R}^2$) the edges of the lower convex hull of a generic lifting of *P* to $\mathbb {R}^3$ (i.e., we add third coordinates to the points in *P* so that no four lifted points are coplanar).

Points in ${\textsf {V} ^\circ }T:=P^\circ \cap {\textsf {V} }T$ are called *inner points* of *T* and points in $P^\circ \setminus {\textsf {V} ^\circ }T$ are called *skipped* in *T* (clearly, for a full triangulation ${\textsf {V} ^\circ }T=P^\circ$ and no points are skipped). Edges in ${\textsf {E} ^\circ }T:=\textsf {E} T\setminus {\textsf {E} _\textsf {hull} }$ are called *inner edges* and edges in ${\textsf {E} _\textsf {hull} }$ are called *boundary edges*. ${{\mathcal {T}}}_{\textsf {full} }(P)$, ${{\mathcal {T}}}_{\textsf {part} }(P)$, and ${{\mathcal {T}}}_{\textsf {reg} }(P)$ will denote the set of all full, partial, and regular triangulations of *P*, respectively.

Every full and every regular triangulation of *P* is also a partial triangulation of *P*. If *P* is in convex position (i.e., ${{\,\mathrm{\textsf {xtr}\mathrm }\,}}{P}=P$), all three notions coincide. It is well known, that there are point sets with non-regular triangulations, [24], see Sect. 10.2 and Fig. 32.

### Definition 1.4

**Fig. 1:**
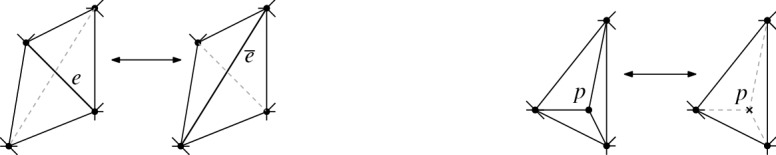
Edge flips and point flips (point removal, left to right; point insertion, right to left)

(*edge flip, point insertion flip, point removal flip*). Let $T \in {{\mathcal {T}}}_{\textsf {part} }(P)$. An edge $e \in {\textsf {E} ^\circ }T$ is called *flippable in* *T* if removing *e* from *T* creates a convex quadrilateral region *Q*. In this case, we denote by *T*[*e*] the triangulation with the other diagonal $\overline{e}$ of *Q* added instead of *e*, i.e., ${\textsf {V} }T[e] := {\textsf {V} }T$ and $\textsf {E} T[e] := \textsf {E} T \setminus \{e\} \cup \{\overline{e}\} = \textsf {E} T \oplus \{e,\overline{e}\}$; we call this an *edge flip*. Occasionally, when we want to emphasize the new edge $\overline{e}$, we will also use the alternative notation $T[\nicefrac {e}{\overline{e}}]$ instead of *T*[*e*], or write $T[e]=T[\nicefrac {e}{\overline{e}}]$. A point $p \in P^\circ$ is called *flippable in* *T* if $p\in P^\circ \setminus {\textsf {V} ^\circ }T$, or if $p \in {\textsf {V} ^\circ }T$ is of degree $3$ in *T*. (a) If $p\in P^\circ \setminus {\textsf {V} ^\circ }T$ then *T*[*p*] is the triangulation with *p* added as a point of degree $3$ (there is a unique way to do so); we call this a *point insertion flip*. (b) If $p\in {\textsf {V} ^\circ }T$ is of degree $3$ in *T* then *T*[*p*] is obtained by removing *p* and its incident edges; we call this a *point removal flip*. See Fig. 1. A *bistellar flip* is one of the three: an edge flip, a point insertion flip, or a point removal flip.

Hence, whenever we write *T*[*x*] for a partial triangulation *T*, then *x* is either a flippable point in $P^\circ$ or a flippable edge in ${\textsf {E} ^\circ }T$. We will use *T*[*x*, *y*] short for (*T*[*x*])[*y*], etc.

### Definition 1.5

(*bistellar flip graph, edge flip graph*). The *bistellar flip graph* of *P* is the graph with vertex set ${{\mathcal {T}}}_{\textsf {part} }(P)$ and edge set $\{\{T,T[x]\} \mid T \in {{\mathcal {T}}}_{\textsf {part} }(P),~x\in P^\circ \cup {\textsf {E} ^\circ }T\ \text {flippable in}\ T \}$. The *edge flip graph* of *P* is the graph with vertex set ${{\mathcal {T}}}_{\textsf {full} }(P)$ and edge set $\{\{T,T[e]\} \mid T \in {{\mathcal {T}}}_{\textsf {full} }(P),~e\in {\textsf {E} ^\circ }T\ \text {flippable in}\ T \}$.

**Fig. 2:**
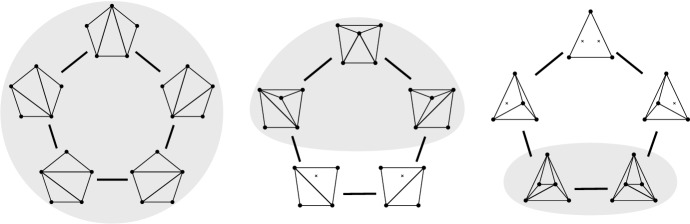
Bistellar flip graphs for five points. Small crosses indicate skipped points in *P*. The shaded areas contain the edge flip graph, the subgraph induced by full triangulations

**Fig. 3:**
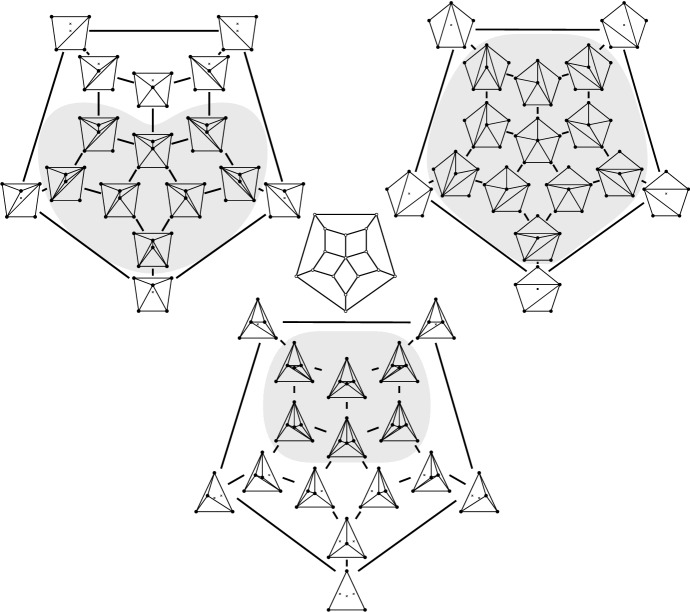
Sets of six points with isomorphic bistellar flip graphs of partial triangulations. The shaded areas cover the edge flip graphs of full triangulations. (Crosses indicate skipped points)

See Figs. 2, 3, and 34 for examples of flip graphs.

### Results—Full Triangulations

The *edge flip graph* is the subgraph of the bistellar flip graph induced by ${{\mathcal {T}}}_{\textsf {full} }(P)$ (see Figs. 2 and 3). Lawson [21] showed that the edge flip graph is connected. A trivial upper bound for the vertex connectivity (see Definition 2.1) of any graph is the minimum vertex degree of the graph; for the edge flip graph this is the minimum number of flippable edges in any triangulation of *P*. This number has been investigated by Hurtado et al. in 1999 [20, Theorem 4.1].

#### Theorem 1.6

[20] Any $T\in {{\mathcal {T}}}_{\textsf {full} }(P)$ has at least $\lceil {n}/{2}-2\rceil$ flippable edges. This bound is tight for all $3\le n\in \mathbb {N}$ (i.e., for every *n* there is a set *P*, $|P|=n$, with a triangulation with exactly $\lceil {n}/{2}-2\rceil$ flippable edges).

We provide a lower bound on the vertex connectivity of the edge flip graph that matches this lower bound of $\lceil {n}/{2}-2\rceil$ for the minimum vertex degree, actually in the refined form $\max {\{\lceil {n}/{2}-2\rceil , h-3\}}$, see Hoffmann et al. [18]. Note, however, that there are sets of points where all full triangulations allow more than $\max {\{\lceil {n}/{2}-2\rceil , h-3\}}$ edge flips. (Consider, e.g., the top left point set in Fig. 3: $n=6$, $h=4$, thus $\max {\{\lceil {n}/{2}-2\rceil , h-3\}}= 1$, but the edge flip graph has minimum vertex degree $2$.) We show that, for *P* large enough, the minimum vertex degree always determines the vertex connectivity of the edge flip graph.

#### Theorem 1.7

(i) There exists $n_0 \in \mathbb {N}$, such that the edge flip graph of any set of $n \ge n_0$ points in general position in the plane is $\delta$-vertex connected, where $\delta$ is the minimum vertex degree in the edge flip graph.
(ii) For $5 \le n \in \mathbb {N}$, the edge flip graph of any set of *n* points with *h* extreme points in general position is $\max {\{\lceil {n}/{2}-2\rceil , h-3\}}$-vertex connected. This is tight: For every $n \in \mathbb {N}$ there is a triangulation of some set of *n* points with no more than $\max {\{\lceil {n}/{2}-2\rceil , h-3\}}$ flippable edges.

Obviously, for $n\ge n_0$, (i) implies (ii). In fact, we do not know whether the restriction “*n* large enough” is required in (i). Still, apart from covering the range to $n_0$, we consider the proof for (ii) of independent interest, since it provides some extra insight to the structure of the edge flip graph via so-called subdivisions, and it is an introduction to the proof for the bistellar flip graph. Although some concrete value of $n_0$ could be easily extracted from the proof, getting a reasonable value is lengthy and tedious, and it is not clear where to stop. Moreover, we believe that the min-degree bound holds for all point sets. This is supported by a recent computer-assisted investigation for small *n* in [12] with the help of the Graz data base of planar order types [1], with the result that for *n* up to 10, the vertex connectivity is determined by the smallest degree of the edge flip graph. This endeavour was nontrivial, given the huge number of order types (14 309 547 for ten points), and for each order type the large number of triangulations (ranging from 250 to 4 550 for ten points).

### Results—Partial Triangulations

The bistellar flip graph is connected, as it follows easily from the connectedness of the edge flip graph, see [24, Sect. 3.4.1]. Here is the counterpart of Theorem 1.6 addressing the minimum vertex degree in the bistellar flip graph, shown by De Loera et al. in 1999 [25, Theorem 2.1].

#### Theorem 1.8

[25] Any $T \in {{\mathcal {T}}}_{\textsf {part} }(P)$ allows at least $n-3$ flips. This bound is tight for all *P*.

Again, we show that the vertex connectivity equals the minimum degree.

#### Theorem 1.9

Let $4 \le n \in \mathbb {N}$. The bistellar flip graph of any set of *n* points in general position in the plane is $(n-3)$-vertex connected. This is tight: Any triangulation of a point set that skips all inner points has degree $n-3$ in the bistellar flip graph (with exactly $h-3$ edge flips and exactly $n-h$ point insertion flips).

This answers (for points in general position) a question mentioned by De Loera, Rambau, and Santos in 2010 [24, Exercise 3.23], and by Lee and Santos in 2017 [22, p. 442]. Before we mention further results, we provide some context. Along the way, we encounter some tools and provide intuition relevant later in the paper.

### Context—Convex Position, Associahedron, and Balinski’s Theorem

**Fig. 4:**
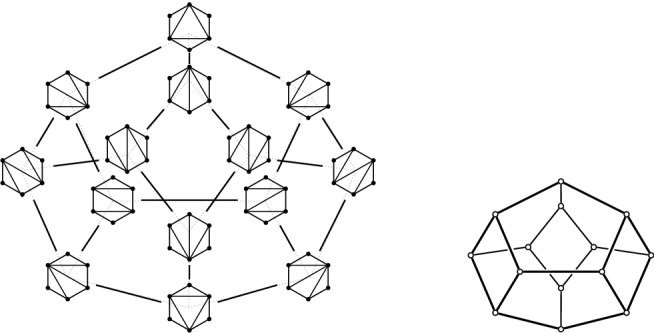
The flip graph of a convex hexagon, the graph of the 3-dimensional associahedron

Suppose *P* is in convex position. Then ${{\mathcal {T}}}_{\textsf {full} }(P)={{\mathcal {T}}}_{\textsf {part} }(P)={{\mathcal {T}}}_{\textsf {reg} }(P)$ is the set of triangulations of a convex *n*-gon whose study goes back to Euler, with one of the first appearances of the Catalan Numbers. There is an $(n-3)$-dimensional convex polytope, the *associahedron*, whose vertices correspond to the triangulations of a convex *n*-gon, and whose edges correspond to edge flips between these triangulations, see [9] for a historical account. That is, the 1-skeleton (graph) of this polytope is isomorphic to the flip graph of *P*, see Fig. 2 (left) for $n=5$ and Fig. 4 for $n=6$. Here Balinski’s Theorem from 1961 comes into play.

#### Theorem 1.10

(Balinski’s Theorem [3]). The 1-skeleton of a convex *d*-dimensional polytope is at least *d*-vertex connected.

**Fig. 5:**
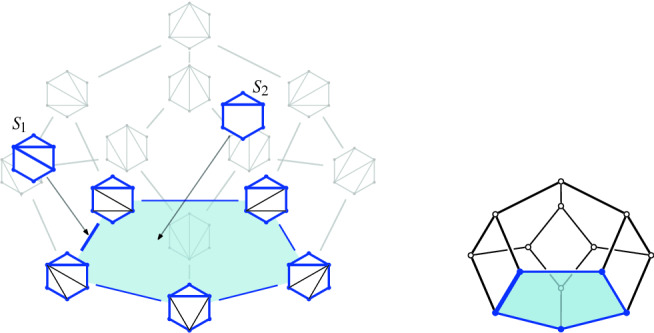
A subdivision $S_1$ with one edge missing representing an edge (1-face) of the 3-dimensional associahedron, and a subdivisions $S_2$ with two edges missing representing a facet (2-face)

Thus we can conclude that the flip graph of *n* points in convex position is $(n-3)$-vertex connected. The face structure of the associahedron is easily explained via so-called subdivisions (also known as polyhedral subdivisions or convex decompositions), which will feature prominently in our arguments. Specialized to the setting of convex position, a subdivision *S* is a plane graph on *P* with all boundary edges present, and some diagonals. We identify every such subdivision with the set of all its possible completions to a triangulation (which we will call *refinements*). Figure 5 illustrates on two examples for $n=6$, that if *S* misses *i* edges towards a triangulation, then its completions to triangulations correspond to the vertices of an *i*-face of the associahedron; in particular, no edge missing (a triangulation) corresponds to a vertex, and one edge missing corresponds to an edge (a flip). This correspondence will guide our intuition, even in more general settings in which there is no polytope in the background.

### Context—Regular Triangulations and Secondary Polytope

All examples of bistellar flip graphs we have seen so far—Figs. 2 and 3, and sets in convex position—are graphs of polytopes, which is not true in general. (Up to six points, there is only one order type which has partial triangulations that are not regular, see Fig. 34 in Sect. 11.) The reason is simply that for the underlying point sets in these examples all partial triangulations are regular. Specifically, there is the following generalization of the associahedron, a highlight of the topic of triangulations from the late eighties due to Gelfand et al. [15] (see [24, Thm. 5.1.9]) (here specialized to points in the plane).

#### Theorem 1.11

(secondary polytope [15]). For every set *P* of *n* points in general position in the plane, there is an $(n-3)$-dimensional polytope $\Sigma \text{- }\textsf {poly} (P)$, the ***secondary polytope*** of *P*, whose 1-skeleton is isomorphic to the bistellar flip graph of ***regular triangulations*** of *P*.

Again, with the help of Balinski’s Theorem (Theorem 1.10), we immediately get: The bistellar flip graph of *regular* triangulations of *P* is $(n - 3)$-vertex connected. Our Theorem 1.9 is the corresponding result for the bistellar flip graph of *all partial* triangulations. Note, however, that it is not a generalization, since it does not imply the result for regular triangulations.

### Context—Simplicial Complex of Plane Graphs and Its Dual Flip Complex

A polytopal representation of the edge flip graph or the bistellar flip graph of all partial triangulations is not known. In fact, a clean result as with the secondary polytope is not possible (see [24] for details). However, there is a construction, due to Orden and Santos [31], of a simple high-dimensional polytope whose 1-skeleton represents all so-called *pseudo-triangulations* of a planar point set and flips between them, following an earlier construction by Rote et al. [32] of a corresponding polytope for *pointed pseudo-triangulations*.

Moreover, the edge flip graph of full triangulations of a planar point set does form the 1-skeleton of a closely related higher-dimensional structure, the *flip complex*, first described by Orden and Santos [31] and later rediscovered by Lubiw et al. [26]. The flip complex is not a polytope, but it is a polytopal complex (informally, a collection of convex polytopes that intersect only in common faces) with a particularly simple topology (it is homotopy equivalent to a ball). For the reader familiar with these notions, we briefly review how our findings fit into this context (for a quick reference for the relevant terminology from topology theory, see, e.g., [5]). This is not essential as a tool for our proofs and the rest of the paper can be followed without this context. Rather, our results shed some extra light on these structures considered in the literature.

Following [26], let us consider

$$\begin{aligned} \mathbb {T} := \mathbb {T}(P) = \left\{ E \subseteq {P \atopwithdelims ()2}\;\big |\;\;\text {no two edges in { E} cross}\right\} , \end{aligned}$$

i.e., these are the edge sets of plane graphs on *P*. Since this family of sets is closed under taking subsets, it is a simplicial complex, and we call its elements *faces* of $\mathbb {T}$. By definition, the *dimension* of a face $F\in \mathbb {T}$ is $\dim F:=|F|-1$. The inclusion-maximal faces (called *facets* of $\mathbb {T}$) are exactly the edge sets of full triangulations of *P*. All facets have equal dimension $m-1$ (where $m:=3n-h-3$, see Lemma 4.6). (Such simplicial complexes with all of their facets having the same dimension are called *pure*.)

In [26], it is shown that $\mathbb {T}$ is shellable and homeomorphic to an $(m-1)$-dimensional ball (this also follows from the results in [31]). This fact was instrumental in the proof of the so-called *orbit conjecture* regarding flips in edge-labeled full triangulations formulated in [7] and proved in [26].

A face *F* of dimension $m-2$ is a plane graph on *P* with exactly one edge missing towards a full triangulation. If *F* is obtained from a full triangulation by removal of a flippable edge, there are exactly two ways to complete it to a triangulation—we call *F* an *interior* $(m-2)$-*face*; otherwise, there is exactly one way to do so—we call *F* a *boundary* $(m-2)$-*face*. A face of any dimension is called a *boundary face* if it is contained in a boundary $(m-2)$-face, and it is called an *interior face*, otherwise (hence, all facets are interior faces). We have the following.

#### Theorem 1.12

[26, Proposition 3.7] $F \in \mathbb {T}$ *is an interior face iff* ${\textsf {E} _\textsf {hull} }\subseteq F$, *and all bounded regions of F are convex* (in other words, iff (*P*, *F*) is what we will call a subdivision, see Definition 4.1 and also discussion in Sect. 1.3).

From the Coarsening Lemma 4.10 in Sect. 4, the following property of $\mathbb {T}$ is implied.

#### Theorem 1.13

Every interior face *F* of  $\mathbb {T}$ contains an interior face of dimension $m +1 - \lceil {n}/{2} \rceil$.

The flip graph can be seen as a structure dual to $\mathbb {T}$, with the vertices of the flip graph corresponding to the facets of $\mathbb {T}$, and two vertices adjacent in the flip graph, if the corresponding facets of $\mathbb {T}$ share (i.e., contain) a common $(m-2)$-face (which is clearly interior). This can be generalized to the flip complex $\mathbb {X}=\mathbb {T}^*$, see [26], with each face of $\mathbb {X}$ (which is dual to an interior face of $\mathbb {T}$) corresponding to a subdivision as we define it below (see Theorem 1.12 above). Each such face is a product of associahedra, and the vertices of such a face of the flip complex are the triangulations refining this subdivision (Definition 4.2). In terms of the flip complex, the Coarsening Lemma 4.10 and Theorem 1.13 can be restated as saying that every inclusion-maximal face of the flip complex is of dimension at least $\max {\{{n}/{2} - 2, h-3\}}$ (see also Theorem 6.1).

### Results—Regular Triangulations

We study the (well-known, see [24]) partially ordered sets of *full and partial subdivisions* of *P*, respectively (see Definitions 4.1 and 7.1), in which triangulations are the minimal elements. We introduce the notions of *slack* of a subdivision (Definitions 4.5 and 7.2), *perfect coarsening* (Definition 8.1) and *perfect coarsener* (Definition 8.2), and we prove the so-called Coarsening Lemmas 4.10 and 8.6 (these can be considered extensions of Theorems 1.6 and 1.8). We consider these notions and lemmas our main contributions besides Theorems 1.7 and 1.9. Together with a sufficient condition for the regularity of partial triangulations and subdivisions (Theorem 10.1 and Regularity Preservation Lemma 10.10), these yield several other results on the structure of flip graphs. In particular, they allow us to settle, in the unexpected direction, a question by Francisco Santos (in personal communication, 2019) regarding the size of certificates for the existence of non-regular triangulations of a given point set in the plane.

#### Theorem 1.14

For all $n \in \mathbb {N}$ there is a set of at least *n* points in general position in the plane, which has non-regular triangulations, and for which any proper subset has only regular triangulations (in other words, only full triangulations of the set can be non-regular).

This should be seen in contrast with the situation in higher dimensions. In large enough dimension, every point set with non-regular triangulations has a subset of bounded size (in the dimension) with non-regular triangulations. This holds since, (a) the vertices of any realization of the cyclic polytope with 12 vertices in dimension 8 has non-regular triangulations [23] (see [24, Sect. 5.5.2]), and (b) every large enough set of points in general position in $\mathbb {R}^8$ has a subset of 12 points which are vertices of a cyclic polytope (this follows from Ramsey’s Theorem [13, 16, 36]).

### Approach

All our vertex connectivity bounds rely on a local variant of Menger’s Theorem, the Local Menger Lemma 2.3. This lemma says that, *assuming connectedness*, in order to show *k*-vertex connectivity, it is enough to show *k* internally vertex-disjoint paths between any two vertices *at distance* $2$. Then, in order to establish the min-degree bound of Theorem 1.7 (i), we explicitly construct the necessary paths between vertices at distance $2$, i.e., triangulations *T*[*e*] and *T*[*f*].

**Fig. 6:**
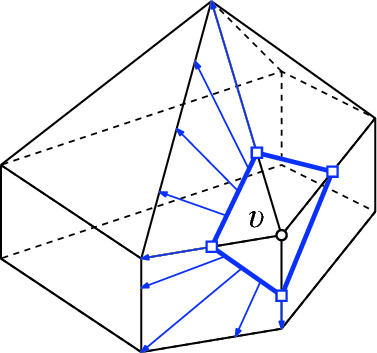
The vertex figure of a polytope, with the mapping of paths in the vertex figure to the 1-skeleton of the polytope indicated

For the bounds in Theorems 1.7 (ii) and 1.9, we look at the neighborhood of a triangulation *T* (which is in one-to-one correspondence with the flippable elements in *T*), supplied with a compatibility relation between the flippable elements (for the edge flip graph, two flippable edges *e* and *f* are compatible, if *e* remains flippable after flipping *f*, Definition 3.6). We call this the link^1^ of *T*, a structure motivated by the *vertex figure* of a vertex in a polytope, see [39, p. 54]: Recall that for a vertex *v* in a *d*-polytope ${{\mathcal {P}}}$, its vertex figure is the $(d-1)$-polytope ${{\mathcal {P}}}'$ obtained by intersecting ${{\mathcal {P}}}$ with a hyperplane that separates *v* from the remaining vertices of the polytope. Vertices of ${{\mathcal {P}}}'$ correspond to edges of ${{\mathcal {P}}}$ incident to *v*, edges in the graph of ${{\mathcal {P}}}'$ correspond to 2-faces of ${{\mathcal {P}}}$ incident to *v*. As indicated in Fig. 6, there is a natural way of mapping paths in the graph of ${{\mathcal {P}}}'$ to paths in the graph of ${{\mathcal {P}}}$. Using the Local Menger Lemma 2.3, this can be easily made an inductive proof of Balinski’s Theorem (Theorem 1.10). We follow exactly this line of thought for flip graphs (where 4- and 5-cycles will play the role of 2-faces), except that we will not need induction: The link of a triangulation avoids 4-cycles *in its complement*, which turns out to directly yield sufficient vertex connectivity (Lemma 2.4).

That is, we borrow intuition from polytope theory, although we know that the edge flip graph and the bistellar flip graph are in general not graphs of polytopes. However, as we will see in Sects. 6 and 11.1, the edge flip graph and the bistellar flip graph can be covered by graphs of $\lceil {n}/{2}-2\rceil$- and $(n-3)$-polytopes, respectively.

It is perhaps worth emphasizing that the arguments we use to prove lower bounds for the vertex connectivity of the flip graphs (in particular, Lemma 2.3) assume the well-known fact that these graphs are always connected—we do not prove this. That is, our techniques will probably *not* help to address the open question whether flip graphs in dimensions 3 and 4 are connected. If at all, they might help to analyze the vertex connectivity of the connected components of flip graphs.

### Paper Organization

In Sect. 2 we show the two lemmas on graph connectivity we mentioned already in Sect. 1.7: The Local Menger Lemma and a lemma about the vertex connectivity of graphs with no 4-cycle in their complement. These may be of independent interest, if only as exercises after teaching Menger’s Theorem in class.

Then the paper splits in three parts, where the second and third part on partial triangulations are largely independent of the first part about full triangulations.

- Section 3 shows the min-degree bound of Theorem 1.7 (i). Section 4 prepares the proof of Theorem 1.7 (ii), which is presented in Sect. 5. We prove the Unoriented Edges Lemma 4.9, which captures the essence of and entails Theorems 1.6 (from [20]) and 1.8 (from [25]) above. It allows us to extend them further for our purposes. Moreover, along the way, we give a short proof of the bound for so-called simultaneously flippable edges by Souvaine et al. [35], and we indicate, how it may help getting insight on so-called *k*-holes in point sets [34] In Sect. 6 we briefly indicate polytopal substructures in the edge flip graph.
- Sections 7–9 show the $(n-3)$-bound in Theorem 1.9.
- Building on the tools developed in Sects. 7–9, in particular the Coarsening Lemma 8.6, Sect. 10 proves a nontrivial new sufficient condition for the regularity of partial triangulations (Theorem 10.1), which can be considered as a generalization of the regularity of stacked triangulations (i.e., triangulations obtained by successively adding points of degree $3$ to a triangulation of ${{\,\mathrm{\textsf {xtr}\mathrm }\,}}{P}$). This will give us a number of implications to be presented in Sect. 11: Covering the bistellar flip graph by graphs of $(n-3)$-polytopes, a characterization of point sets for which all partial triangulations are regular, and, finally, Theorem 1.14 on the size of certificates for the existence of non-regular triangulations in ${{\mathcal {T}}}_{\textsf {part} }(P)$.

We conclude by discussing open problems in Sect. 12.

## Graph Connectivity

We follow the graph theory books of Bollobás [6] and Diestel [10].

### Definition 2.1

For $k \in \mathbb {N}$, a simple undirected graph *G* is *k*-*vertex connected* if *G* is connected, has at least $k+1$ vertices, and removing any set of at most $k-1$ vertices (and their incident edges) leaves the graph connected.

Note that a graph is 1-vertex connected iff it is connected and has at least two vertices. Here is a classical result due to Menger from 1927 [29], see [6, Theorem III.5].

### Theorem 2.2

(Menger’s Theorem [29]). Let *u* and *v* be distinct nonadjacent vertices of a graph *G*. Then the minimal number of vertices separating *u* from *v* is equal to the maximal number of internally vertex-disjoint *u*-*v* paths.

### Convention

From now on, we will use *vertex-disjoint* short for “internally vertex-disjoint.”

We will need a local variant of Menger’s Theorem.

### Lemma 2.3

(Local Menger). Let $k\in \mathbb {N}$ and let *G* be a simple undirected graph. Assume that *G* is *connected*. Then *G* is *k*-vertex connected iff *G* has at least $k+1$ vertices and for any pair of vertices *u* and *v* *at distance* $2$ there are *k* pairwise vertex-disjoint *u*-*v* paths.

### Proof

The direction $(\Rightarrow )$ follows from Menger’s Theorem 2.2. For $(\Leftarrow )$, suppose that for any pair *u* and *v* of vertices at distance 2 there are *k* pairwise vertex-disjoint *u*-*v* paths. We show that no pair of vertices *x* and *y* can be separated by removal of a set $V'$ of at most $k-1$ vertices (different from *x* and *y*). This is true for *x* and *y* adjacent. If *x* and *y* are not adjacent, let $(x=x_0,x_1,\ldots ,x_\ell =y)$ be an *x*-*y* path which uses the minimal number of vertices in $V'$, and among those, a shortest such path (hence $\{x_{i-1},x_{i+1}\} \notin \textsf {E} G$ for $0< i <\ell$). If no vertex on this path is in $V'$ we are done. Otherwise, consider $x_i \in V'$, $0<i<\ell$. We can replace the subpath $(x_{i-1},x_i,x_{i+1})$ by an $x_{i-1}$-$x_{i+1}$ path using none of the vertices in $V'$ as internal vertices ($x_{i-1}$ and $x_{i+1}$ have distance $2$ and hence such a path exists, since there are *k* vertex-disjoint $x_{i-1}$-$x_{i+1}$ paths and $|V'|<k$). We obtain an *x*-*y* walk with one less overlap with $V'$, and we can turn this into an *x*-*y* path with less overlap with $V'$; contradiction. (A *walk* is a path with repetitions of vertices allowed.) $\square$

For the proofs of Theorems 1.7(ii) and 1.9, here is a special property of a graph that guarantees that the minimum vertex degree determines exactly the vertex connectivity. As briefly discussed in Sect. 1.7, we will apply this lemma not directly to the flip graph, but rather to the link, a neighborhood structure of a triangulation.

### Lemma 2.4

Let *G* be a graph with its complement having no cycle of length $4$, i.e., for any sequence $(x_0,x_1,x_2,x_3)$ of four distinct vertices in *G* there exists $i\in \{0,1,2,3\}$ with $\{x_i, x_{i+1~mod ~4}\}\in \textsf {E} G$. Then *G* is $\delta$-vertex connected, where $\delta$ is the minimum vertex-degree in *G*.

### Proof

It suffices to show that for any two distinct nonadjacent vertices *u* and *v* there are $\delta$ vertex-disjoint *u*-*v* paths. Let $z_1,z_2,\ldots ,z_\ell$ be the set of vertices adjacent both to *u* and to *v*. This gives *u*-*v* paths $(u,z_i,v)$, $1 \le i \le \ell$. If $\ell \ge \delta$ we are done. Otherwise, there are vertices $x_{\ell +1},\ldots ,x_\delta$ adjacent to *u* and not to *y*, and there are vertices $y_{\ell +1},\ldots , y_\delta$ adjacent to *v* and not to *u*. For $\ell <j\le \delta$ consider the sequence $(u,y_j,x_j,v)$: None of $\{u,y_j\}$, $\{x_j,v\}$, and $\{u,v\}$ are edges in *G*, hence $\{y_j,x_j\}\in \textsf {E} G$ and $(u,x_j,y_j,v)$ is a *u*-*v* path. That is, we have found another $\delta -\ell$ paths connecting *u* to *v*. All paths constructed are easily seen to be vertex-disjoint. $\square$

## Min-Degree Bound for Full Triangulations

### Convention

In Sects. 3–6 we will mostly use *triangulation* short for “full triangulation.”

In this section we prove Theorem 1.7 (i) (Sect. 3.5) via the following lemma (and the Local Menger Lemma 2.3).

### Lemma 3.1

There exists $n_0 \in \mathbb {N}$, such that any set *P* with $|P| \ge n_0$ has the following property: If *T*[*e*] and *T*[*f*] are distinct triangulations obtained from $T \in {{\mathcal {T}}}_{\textsf {full} }(P)$ by flipping edges *e* and *f*, respectively, then there are $\delta '$ vertex-disjoint *T*[*e*]-*T*[*f*] paths, with $\delta '$ the minimum degree of the two vertices *T*[*e*] and *T*[*f*] in the edge flip graph of *P*.

For the proof of Lemma 3.1 (see Sect. 3.4), we need a better understanding of how flippable edges interact. This will exhibit short cycles, more concretely, 4- or 5-cycles in the edge flip graph (called elementary cycles in [26]). Subpaths of these short cycles will be the building blocks for the *T*[*e*]-*T*[*f*] paths as claimed in Lemma 3.1.

### Basic Terminology

#### Definition 3.2

**Fig. 7:**
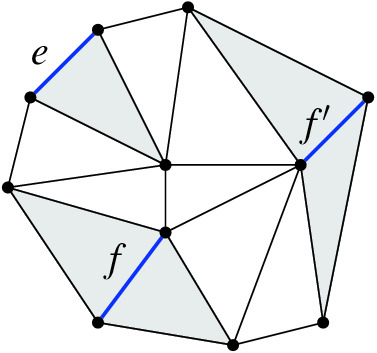
Edges with their territories, which are open triangles or open quadrilaterals. The boundary edge *e* has a triangular territory, and the inner edges *f* and $f'$ have quadrilateral territories

(*territory of an edge*). For $T \in {{\mathcal {T}}}_{\textsf {full} }(P)$ and $e \in \textsf {E} T$, we define the *territory of e*, ${{\,\mathrm{\textsf {terr}\mathrm }\,}}{e} = \textsf {terr} _{T}e$, as the *interior* of the closure of the union of its one or two incident regions in *T*, see Fig. 7. (Recall that the unbounded face of *T* is not a region, see Definition 1.2.)

If *e* is a boundary edge, ${{\,\mathrm{\textsf {terr}\mathrm }\,}}{e}$ is an open triangle (one of the regions of *T*). Otherwise, for *e* an inner edge, it is an open quadrilateral. An inner edge *e* is flippable in *T* iff ${{\,\mathrm{\textsf {terr}\mathrm }\,}}{e}$ is convex.

We can observe right away that if ${{\,\mathrm{\textsf {terr}\mathrm }\,}}{e}$ and ${{\,\mathrm{\textsf {terr}\mathrm }\,}}{f}$ are disjoint for flippable edges in *T*, then we can flip *e* and *f* in any order leading to the same triangulation, i.e., $T[e,f] = T[f,e]$ and (*T*, *T*[*e*], *T*[*e*, *f*], *T*[*f*]) is a 4-cycle in the edge flip graph.

### Two Consecutive Flips

#### Lemma 3.3

Let $T \in {{\mathcal {T}}}_{\textsf {full} }(P)$ and let *e* be a flippable edge in *T* with $T[e] = T[\nicefrac {e}{\overline{e}}]$ (notation as introduced in Definition 1.4). Then:

(i) $$\textsf {E} T \oplus \textsf {E} T[e] = \{e,\overline{e}\}$$.
(ii) For *f* an edge flippable in *T*[*e*] with $T[e,f] =(T[e])[\nicefrac {f}{\overline{f}}]$, we have $T[e,f]=T$, if $f=\overline{e}$, or
  1 $$\begin{aligned} \textsf {E} T \oplus \textsf {E} T[e,f]&=\{e,\overline{e},f,\overline{f}\},\quad \ \text {and} \end{aligned}$$
  2 $$\begin{aligned} \textsf {E} T \setminus \textsf {E} T[e,f]&=\{e,f\}, \end{aligned}$$
  otherwise.

#### Proof

(i) is immediate by definition. For (ii) we observe that $\textsf {E} T \oplus \textsf {E} T[e,f] = \textsf {E} T \oplus \textsf {E} T[e] \oplus \textsf {E} T[e] \oplus \textsf {E} T[e,f]= \{e,\overline{e}\} \oplus \{f,\overline{f}\}$. If $f=\overline{e}$, then $\overline{f} = e$ and we are done. Otherwise, for (1) it is left to show $\{e,\overline{e}\}\cap \{f,\overline{f}\}=\emptyset$. We have $f \ne e$ since $e\notin \textsf {E} T[e]$, we have $f\ne \overline{e}$ by assumption, we have $\overline{f}\ne \overline{e}$ since $\overline{e}\in \textsf {E} T[e]$, and we have $\overline{f} \ne e$ since *e* crosses $\overline{e}$ which is present in *T*[*e*, *f*] (by assumption $f\ne \overline{e}$). Finally, (2) follows from (1), from $e,f \in \textsf {E} T$ (by assumption $f\ne \overline{e}$), and from $|\textsf {E} T|=|\textsf {E} T[e,f]|$. $\square$

Thus, $|{\textsf {E} T \oplus \textsf {E} T[e]}| = 2$ and $|{\textsf {E} T \oplus \textsf {E} T[e,f]}|=4$, unless $T[e,f]=T$. This directly implies

#### Corollary 3.4

The edge flip graph of *P* is triangle-free.

### Interplay of Two Flippable Edges

Here comes an essential lemma about the interplay of two flippable edges in a triangulation.

#### Lemma 3.5

Let *e* and *f* be two distinct edges both of which are flippable in $T \in {{\mathcal {T}}}_{\textsf {full} }(P)$. Then *e* is flippable in *T*[*f*] iff *f* is flippable in *T*[*e*].

#### Proof

We distinguish three cases, depending on the shape of ${{\,\mathrm{\textsf {terr}\mathrm }\,}}{e}\cup {{\,\mathrm{\textsf {terr}\mathrm }\,}}{f}$. This can be composed of two disjoint quadrilaterals (recall that territories are open sets), or it is a pentagon, if *e* and *f* are incident to a common region of *T*.

**Fig. 8:**
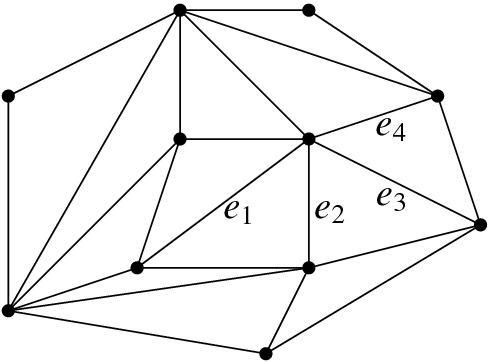
Edges $e_1$ and $e_3$ are independently flippable, edges $e_2$ and $e_3$ are weakly independently flippable, and edges $e_3$ and $e_4$ are dependently flippable

- If ${{\,\mathrm{\textsf {terr}\mathrm }\,}}{e} \cap {{\,\mathrm{\textsf {terr}\mathrm }\,}}{f} = \emptyset$ then, as observed above, *e* is flippable in *T*[*f*] and *f* is flippable in *T*[*e*] (see, e.g., $e_1$ and $e_3$, or $e_1$ and $e_4$ in Fig. 8).
- If ${{\,\mathrm{\textsf {terr}\mathrm }\,}}{e} \cup {{\,\mathrm{\textsf {terr}\mathrm }\,}}{f}$ is a convex pentagon, then *e* is flippable in *T*[*f*] and *f* is flippable in *T*[*e*] (see, e.g., $e_2$ and $e_3$ in Fig. 8).
- We are left with the case of ${{\,\mathrm{\textsf {terr}\mathrm }\,}}{e}\cup {{\,\mathrm{\textsf {terr}\mathrm }\,}}{f}$ a nonconvex pentagon (see, e.g., $e_3$ and $e_4$ in Fig. 8). Let *p* be a reflex vertex in this pentagon. *e* or *f* have to be incident to *p*, otherwise *T* has a reflex vertex in one of its regions. If only one of *e* and *f* is incident to *p*, say *e*, then $\textsf {terr} _{T}e$ is not convex and *e* is not flippable. Hence, *both* *e* and *f* are incident to *p*. But after flipping *e*, the other edge *f* is left “alone” at this vertex *p*, i.e., $\textsf {terr} _{T[e]}f$ is not convex and thus not flippable in *T*[*e*]; similarly, after flipping *f*, edge *e* is not flippable. That is, *e* is not flippable in *T*[*f*] and *f* is not flippable in *T*[*e*].

$$\square $$

In short, the lemma states that, provided *e* and *f* are flippable in *T*, *T*[*e*, *f*] is defined iff *T*[*f*, *e*] is defined. If *T*[*e*, *f*] and *T*[*f*, *e*] are defined, they may be equal or not. With this in mind, we give the following definition (Fig. 8).

#### Definition 3.6

Let $T\in {{\mathcal {T}}}_{\textsf {full} }(P)$ and let *e* and *f* be flippable edges in $\textsf {E} T$ with $e \ne f$.

(i) *e* and *f* are called *independently flippable in* *T* if *e* is flippable in *T*[*f*], and $T[e,f]=T[f,e]$.
(ii) *e* and *f* are called *weakly independently flippable in* *T* if *e* is flippable in *T*[*f*], and $T[e,f] \ne T[f,e]$.
(iii) *e* and *f* are called *compatible in* *T* if *e* and *f* are either independently or weakly independently flippable in *T*.
(iv) *e* and *f* are called *dependently flippable in* *T* if *e* is not flippable in *T*[*f*].

Lemma 3.5 shows that all relations in Definition 3.6 are symmetric.

#### Lemma 3.7

**Fig. 9:**
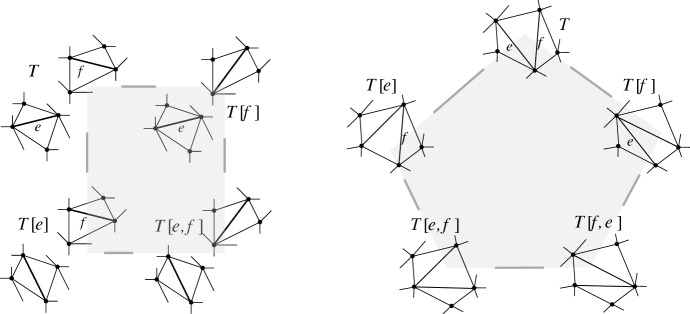
4-cycle (*T*, *T*[*e*], *T*[*e*, *f*], *T*[*f*], *T*) and 5-cycle (*T*, *T*[*e*], *T*[*e*, *f*], *T*[*f*, *e*], *T*[*f*], *T*) for *e* and *f* independently flippable and weakly independently flippable, respectively

Let $T\in {{\mathcal {T}}}_{\textsf {full} }(P)$ and let *e* and $f\ne e$ be flippable edges in *T*. Then:

(i) *e* and *f* are independently flippable
  $$\Leftrightarrow $$ $${{\,\mathrm{\textsf {terr}\mathrm }\,}}{e} \cap {{\,\mathrm{\textsf {terr}\mathrm }\,}}{f} = \emptyset $$
  $\Leftrightarrow$ (*T*, *T*[*e*], *T*[*e*, *f*], *T*[*f*], *T*) is an induced 4-cycle in the edge flip graph of *P*, see Fig. 9, left.
(ii) *e* and *f* are weakly independently flippable
  $\Leftrightarrow$ ${{\,\mathrm{\textsf {terr}\mathrm }\,}}{e} \cup {{\,\mathrm{\textsf {terr}\mathrm }\,}}{f}$ is a convex pentagon
  $\Leftrightarrow$ (*T*, *T*[*e*], *T*[*e*, *f*], *T*[*f*, *e*], *T*[*f*], *T*) is an induced 5-cycle in the edge flip graph of *P*, see Fig. 9, right.

#### Proof

The proof of Lemma 3.5 has identified three disjoint cases for *e* and *f* flippable in *T*. (a) “${{\,\mathrm{\textsf {terr}\mathrm }\,}}{e}\cap {{\,\mathrm{\textsf {terr}\mathrm }\,}}{f} = \emptyset$” immediately yields the three properties listed under (i). (b) “${{\,\mathrm{\textsf {terr}\mathrm }\,}}{e} \cup {{\,\mathrm{\textsf {terr}\mathrm }\,}}{f}$ is a convex pentagon” implies the three properties listed under (ii), and (c) “${{\,\mathrm{\textsf {terr}\mathrm }\,}}{e} \cup {{\,\mathrm{\textsf {terr}\mathrm }\,}}{f}$ is a nonconvex pentagon” contradicts all conditions in (i) and (ii), since we have shown that *e* is not flippable in *T*[*f*] and *f* is not flippable in *T*[*e*] in that case. The 4- and 5-cycles have to be induced since the edge flip graph is triangle-free (Corollary 3.4). $\square$

### Proof of Lemma 3.1

#### Proof

Let *e* and $f\ne e$ be flippable edges in *T*, i.e., $T[e] = T[\nicefrac {e}{\overline{e}}]$ and $T[f] = T[\nicefrac {f}{\overline{f}}]$ are defined for appropriate edges $\overline{e}$ and $\overline{f}$. Suppose the degree of *T*[*e*] is at most the degree of *T*[*f*]. Our task is to identify, for each edge *g* flippable in *T*[*e*], a path $(T[e],T[e,g], \ldots , T[f,g^*],T[f])$, with these paths required to be vertex-disjoint. For this we distinguish four cases, depending on *g*.

- $$g=\overline{e}$$: (*T*[*e*], *T*, *T*[*f*]) (note $$T = T[e,\overline{e}]$$).
- $g=f$, if *f* is flippable in *T*[*e*]: (*T*[*e*], *T*[*e*, *f*], *T*[*f*]) or $(T[e], T[e,f],T[f,e], T[f])$.
- *g* is flippable in *T*[*e*], *T*, and *T*[*f*]:        $$\qquad \qquad \qquad \displaystyle \left( T[e],\begin{array}{c}T[e,g] \text{ or }\\ T[e,g], T[g,e]\end{array},~T[g], \begin{array}{c}T[g,f] \text{ or }\\ T[g,f], T[f,g]\end{array},~T[f]\right) $$.
- We still miss the paths $(T[e], T[e,g], \ldots )$ for edges $g \notin \{\overline{e},f\}$ flippable in *T*[*e*] but not flippable in *T* or not flippable in *T*[*f*]. There are at most eight such edges *g*, since for this to happen, *g* must be an edge of $\textsf {terr} _{T}e=\textsf {terr} _{T[e]}\overline{e}$ or of $\textsf {terr} _{T}f$. For such an edge *g* choose an edge $g^*\notin \{\overline{f},e\}$ flippable in *T*[*f*] but not flippable in *T* or *T*[*e*]. Because of our degree condition, $g^*$ must exist. Note that *g* may equal $g^*$, if *g* is flippable in *T*[*f*] but not in *T*. Now we choose edges *x* and *y* flippable in *T* with the three sets
  $$\begin{aligned} \textsf {terr} _{T}x,\quad \textsf {terr} _{T}y,\ \ \text {~and~}\ \ \textsf {terr} _{T}e \cup \textsf {terr} _{T}f \cup \textsf {terr} _{T}g \cup \textsf {terr} _{T}g^* \end{aligned}$$
  pairwise disjoint. These conditions allow for the following path:
  $$\begin{aligned} (T[e],T[e,g],T[e,g,x], T[e,g,x,y],T[e,x,y],T[x,y],\quad&\\ T[f,x,y], T[f,g^*,x,y], T[f,g^*,x], T[f,g^*],T[f])&. \end{aligned}$$
  Every *g* in this final category is paired up with a different $g^*$ and a different pair $\{x,y\}$ is chosen for building such a *T*[*e*]-*T*[*f*] path. *P* large enough will allow us to do so, by Theorem 1.6 and since we have to deal only with at most a constant (at most eight) such cases.

For vertex-disjointness, we define for $T'$ an internal vertex on such a *T*[*e*]-*T*[*f*] path the *signature* $(\textsf {E} T[e] \cap \textsf {E} T[f])\setminus \textsf {E} T'$. The internal vertices of our long paths in (d) have signatures

$$\begin{aligned} (\{g\},\{g,x\},\{g,x,y\},\{x,y\},\{x,y\},\{x,y\},\{g^*,x,y\},\{g^*,x\},\{g^*\}) \end{aligned}$$

while previous cases gave signatures (again of internal vertices)

$$\begin{aligned} \underbrace{(\emptyset )}_{\mathrm {(a)}\ g=\overline{e}},\quad \underbrace{(\emptyset ) \text { or }(\emptyset ,\emptyset )}_{\mathrm {(b)}\ g=f},\quad \text {or}\underbrace{(\{g\}, \{g\}, \ldots , \{g\}).}_{\mathrm {(c)}\ g\ \text {compatible with}\ e\ \text {and}\ f\ \text {in}\ T} \end{aligned}$$

The vertex-disjointness of these paths can be readily concluded from these sequences and the proof is completed. $\square$

**Fig. 10:**
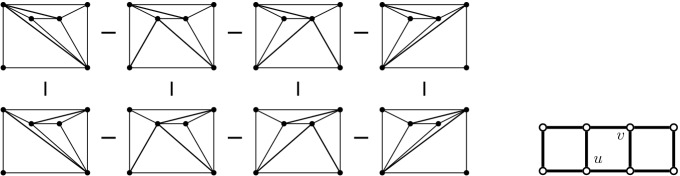
An edge flip graph with two vertices *u* and *v* of degree $3$ at distance $2$, which do not allow three vertex-disjoint connecting paths

The restriction “*P* large enough” is essential in Lemma 3.1, see Fig. 10.

### Proof of Theorem 1.7 (i)

#### Proof

The edge flip graph is connected and triangulations $T'$ and $T''$ at distance $2$ in the edge flip graph can be written as $T' = T[e]$ and $T'' = T[f]$, for some $T \in {{\mathcal {T}}}_{\textsf {full} }(P)$ and $e,f \in \textsf {E} T$. Therefore, Lemma 3.1 indeed implies Theorem 1.7 (i) by the Local Menger Lemma 2.3. $\square$

As mentioned before, while “*P* large enough” is essential in Lemma 3.1, we do not know whether it can be removed in Theorem 1.7 (i).

## Coarsening Full Subdivisions

In preparation of the proof of Theorem 1.7 (ii), which will be presented in Sect. 5, we introduce full subdivisions of a point set, which form a partially ordered set under a relation we call refinement, and slack, a parameter of subdivisions. As mentioned when discussing the flip complex in Sect. 1.5 above, subdivisions correspond to the faces of the flip-complex. The refinement partial order corresponds to inclusion of one face in another, and the slack of a subdivision is equal to the dimension of the corresponding face of the flip complex. This will allow us to prove a lower bound on how many edges are compatible with a given flippable edge in a triangulation.

### Full Subdivisions

#### Definition 4.1

(*full subdivision*). A *full subdivision* *S* of *P* is a connected plane graph with ${\textsf {V} }S = P$ and ${\textsf {E} _\textsf {hull} }\subseteq \textsf {E} S$, and all regions of *S* convex.

#### Convention

In Sect. 4 up to Sect. 6 we will use ***subdivision*** short for “full subdivision.”

#### Definition 4.2

(*coarsening, refinement*).

- Given subdivisions $S_1$ and $S_2$ of *P*, we call $S_1$ a *refinement of* $S_2$, in symbols $S_1 \preceq S_2$, if $\textsf {E} S_1\supseteq \textsf {E} S_2$ (or $S_2$ a *coarsening of* $S_1$, in symbols $S_2 \succeq S_1$).
- Given a subdivision *S* of *P*, we let ${{\mathcal {T}}}_{\textsf {full} }{\langle S \rangle } := \{ T \in {{\mathcal {T}}}_{\textsf {full} }(P)\mid T \preceq S\}$, the set of triangulations of *P* refining *S*.

(In the literature, triangulations in ${{\mathcal {T}}}_{\textsf {full} }{\langle S \rangle }$ are sometimes also called *S*-constrained triangulations of *P*.) Obviously, $\preceq$ is a partial order on subdivisions of *P* with the triangulations the minimal elements. Note that if $S_1 \preceq S_2$, then every region of $S_1$ is contained in some region of $S_2$, hence the name “refinement.” Here is a notion that allows us to identify edges in a subdivision that can be individually removed (not necessarily simultaneously removed) in order to obtain a coarsening.

**Fig. 11:**
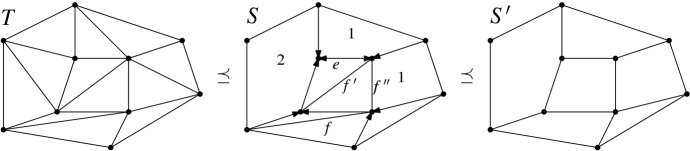
A subdivision *S*, with edges directed to endpoints where they are locked. Edge *e* is locked at both endpoints. Edges *f*, $f'$, and $f''$ are not locked, each one can be individually removed obtaining a coarsening subdivision. Slacks of non-triangular regions are indicated (triangular regions have slack 0). ${{\,\mathrm{\textsf {sl}\mathrm }\,}}{S}=2+1+1=4$. Removal of *f* and $f'$ yields a $\preceq$-maximal subdivision $S'$ coarsening *S*. *T* is a triangulation refining *S*

#### Definition 4.3

(*locked*). In a graph *G* on *P*, an edge $e \in \textsf {E} G$ is *locked at endpoint* *p* if the angle obtained at *p* (between the edges adjacent to *e* in radial order around *p*) after removal of *e* is at least $\pi$ (in particular, if *p* has degree $1$ or $2$ in *G*). See Fig. 11.

An edge in a triangulation is flippable iff it is not locked at any endpoint (in a triangulation, an edge can be locked at at most one of its endpoints). If *S* is a subdivision, then removal of an edge *e* in *S* gives a subdivision iff *e* is locked at none of its endpoints in *S* (here, for a subdivision, an edge can be locked at both of its endpoints). Hence, the $\preceq$-maximal subdvisions are those with all of their edges locked. Here are some simple fundamental properties of locking.

#### Observation 4.4

Let *G* be a graph on *P* and $p \in {\textsf {V} }G$.

(i) Any two edges locked at *p* must be consecutive in the radial order around *p*.
(ii) There are at most two edges locked at *p*, unless *p* has degree $3$ in *G*.
(iii) If *G* is a subdivision and $p \in P^\circ$ is of degree $3$, then the three edges incident to *p* are locked at *p*.

#### Definition 4.5

(*slack, refined slack*). Let *r* be a region of a subdivision *S*, a convex set bounded by a *k*-gon, $k \ge 3$. Then we define the *slack*, ${{\,\mathrm{\textsf {sl}\mathrm }\,}}{r}$, of *r* as $k-3$, and the *refined slack*, $\textsf {sl} ^*r$, of *r* as $\lceil {{{\,\mathrm{\textsf {sl}\mathrm }\,}}{r}}/{2} \rceil$. See Fig. 11. The *slack*, ${{\,\mathrm{\textsf {sl}\mathrm }\,}}{S}$, of subdivision *S* is the sum of the slacks of its regions. The *refined slack*, $\textsf {sl} ^*S$, is the sum of the refined slacks of its regions.

Note that $k-3$ is the number of edges it takes to triangulate a *k*-gon. Hence, the slack of a subdivision is the number of edges which have to be added towards a triangulation. The refined slack $\lceil ({k-3})/{2} \rceil = \lfloor {k}/{2} \rfloor - 1$ of a *k*-gon cannot be easily motivated at this point. It will become apparent only in the proof of Lemma 4.9 (ii) where it is used as the excess (relative to a triangle) of the largest independent set of a *k*-cycle. We need the following well-known facts on the number of edges and regions of triangulations.

#### Lemma 4.6

For $T \in {{\mathcal {T}}}_{\textsf {full} }(P)$, the number of edges, $|\textsf {E} T|$, equals $3n-3-h= 3n^{\circ }-3+2h$ and the number of regions, $|{\textsf {R} }T|$, equals $2n-2-h= 2n^{\circ }-2+h$ (recall that the unbounded face is not a region).

#### Observation 4.7

For a subdivision *S*, we have

$$\begin{aligned} {{\,\mathrm{\textsf {sl}\mathrm }\,}}{S}&=3n-3-h- |\textsf {E} S| = 3n^\circ - 3 + h - |{\textsf {E} ^\circ }S|\quad \ \text {and}\\&=2n-2 -h -|{\textsf {R} }S| = 2n^\circ -2+h -|{\textsf {R} }S|. \end{aligned}$$

### Unoriented Edges Lemma

#### Definition 4.8

(*partially oriented graph, well-oriented graph*). Let $\vec {G}$ be a graph *G* on *P* with *some* (not necessarily all) of its edges oriented to *one* (not both) of its endpoints and some edges unoriented; we call this a *partially oriented graph*. $\vec {G}$ is *well oriented* if (a) no edge is directed to a point in ${{\,\mathrm{\textsf {xtr}\mathrm }\,}}{P}$, and (b) if edges *e* and *f* are directed to a common point *p*, then *e* and *f* have to be consecutive in radial order around *p*.

Note right away that condition (b) for a well-oriented graph shows that no point in such a graph can have indegree more than $3$, and indegree $3$ is possible only for points of degree $3$. Moreover, given a subdivision, suppose we orient each locked *inner* edge to one locking endpoint (and we leave all other edges, in particular the boundary edges, unoriented), then Observation 4.4 shows that this is a well-oriented graph (hence, *S* in Fig. 11, center, serves as an example of a well-oriented graph). This is how we will later employ the following lemma.

#### Lemma 4.9

(Unoriented Edges Lemma). Let $\vec {S}$ be a partially oriented subdivision of *P*. Let $C_i$, $i \in \mathbb {N}_0$, be the number of inner points of $\vec {S}$ with indegree *i* and let $D:={{\,\mathrm{\textsf {sl}\mathrm }\,}}{S}$ and $D^* := \textsf {sl} ^*S$.

(i) If $C_i = 0$ for $i \ge 4$ then the number of unoriented inner edges equals
  $$\begin{aligned} n-3 - C_3 - D +(C_1+2C_0) \ge n-3 - C_3 - D \ge h-3-D. \end{aligned}$$
(ii) Suppose $\vec {S}$ is well oriented. Then the number of unoriented inner edges is at least
  3 $$\begin{aligned} \frac{n}{2} - 2 - \frac{D+D^*}{2}\ge \frac{n}{2} - 2 - D. \end{aligned}$$

#### Proof

(i) $n^{\circ }= C_3 + C_2 +C_1 +C_0$ since $C_i = 0$ for $i \ge 4$. $|{\textsf {E} ^\circ }S|=3n^{\circ }- 3 + h -D$ (Observation 4.7). Since $\vec {S}$ has $3C_3 +2 C_2 + C_1$ oriented edges, the number of unoriented inner edges is 4
(ii) Let every inner point charge $1$ to each region incident to it that lies between two incoming edges. The overall charge made is exactly $3 C_3 + C_2$ (if the indegree is $3$, the degree is $3$; if the indegree is $2$, the two incoming edges are consecutive). While each triangular region can be charged at most once, other regions can be multiply charged: A region with slack *d*, a $(d+3)$-gon, is charged at most $\lfloor (d+3)/2\rfloor = 1 + \lfloor (d+1)/2\rfloor = 1 + \lceil d/2\rceil$ times (vertices charging a region *r* cannot be consecutive along the boundary of *r*). Hence, if $\ell$ is the number of regions charged at all, the overall charge made to these $\ell$ regions is at most $\ell + D^*$. Thus $3 C_3 + C_2 \le \ell + D^*$, i.e., $\ell$ is at least $3 C_3 + C_2 - D^*$. Moreover, $\ell$ is at most $2 n^{\circ }- 2 + h - D$, the overall number of regions (Observation 4.7). That is,
  $$\begin{aligned} 3 C_3 + C_2 - D^*&\le 2 n^{\circ }- 2 + h - D=\underbrace{n^{\circ }+ h}_{=n} -2 +\overbrace{C_3 + C_2 +C_1 +C_0}^{n^{\circ }=} - D\\ \Longleftrightarrow \quad C_3&\le \frac{n}{2} - 1 - \frac{D-D^*}{2} +\frac{C_0 + C_1}{2}. \end{aligned}$$

We plug this bound on $C_3$ into the number of unoriented edges derived in (4) above:

$$\begin{aligned}&n-3 - C_3 -D +C_1+2C_0\\&\qquad \ge n-3- \frac{n}{2} + 1 + \frac{D-D^*}{2} - \frac{C_0 + C_1}{2}-D +C_1+2C_0\\&\qquad =\frac{n}{2} - 2 - \frac{D+D^*}{2} + \frac{C_1 + 3 C_0}{2}. \end{aligned}$$

The left bound in (ii) on the number of unoriented follows readily. Moreover, $\lceil {d}/{2}\rceil \le d$ for $d \in \mathbb {N}_0$, hence $D^* \le D$, i.e., $({D+D^*})/{2} \le D$ and the right bound in (ii) is implied. $\square$

#### Lemma 4.10

(Coarsening Lemma for full subdivisions). Any $\preceq$-maximal subdivision *S* of *P* (i.e., all edges in ${\textsf {E} ^\circ }S$ are locked) has slack at least $\max {\{n/2 - 2, h-3\}}$.

#### Proof

Orient each edge in *S* to a locking endpoint (ties broken arbitrarily). This gives a well-oriented graph $\vec {S}$ without unoriented inner edges. Since Lemma 4.9, (i) and (ii), guarantees at least $\max {\{h-3- {{\,\mathrm{\textsf {sl}\mathrm }\,}}{S},{n}/{2} - 2- {{\,\mathrm{\textsf {sl}\mathrm }\,}}{S}\}}$ unoriented inner edges, we have $\max {\{{n}/{2} - 2,h-3\}} - {{\,\mathrm{\textsf {sl}\mathrm }\,}}{S} \le 0$. $\square$

Here is the essential implication on the number of compatible edges.

#### Corollary 4.11

(i) Every $T \in {{\mathcal {T}}}_{\textsf {full} }(P)$ has at least $\max {\{{n}/{2} - 2,h-3\}}$ flippable edges.
(ii) Given $T \in {{\mathcal {T}}}_{\textsf {full} }(P)$ and *e* flippable in *T*, there are at least $\max {\{{n}/{2} - 3,h-4\}}$ edges in $\textsf {E} T$ compatible with *e*.

#### Proof

(i) Let *S* be a $\preceq$-maximal subdivision with $S \succeq T$. Then all edges in $\textsf {E} T \setminus \textsf {E} S$ are flippable in *T*. Since $|\textsf {E} T \setminus \textsf {E} S| = {{\,\mathrm{\textsf {sl}\mathrm }\,}}{S}$, the claim follows by Lemma 4.10.
(ii) Let $T_{-e}$ be the graph obtained by removing the edge *e* from *T*. Since *e* is flippable, $T_{-e}$ is a subdivision. Let *S* be a $\preceq$-maximal coarsening of $T_{-e}$. All edges in $\textsf {E} T_{-e} \setminus \textsf {E} S$ are compatible with *e* in *T*. Since $|\textsf {E} T_{-e} \setminus \textsf {E} S| = {{\,\mathrm{\textsf {sl}\mathrm }\,}}{S}-1$, the claim follows again by Lemma 4.10. $\square$

We see that Corollary 4.11 (i) is Theorem 1.6 by Hurtado et al. [20]. Actually, the set $\textsf {E} T \setminus \textsf {E} S$ in the argument for Corollary 4.11(i) is what is called *ps-flippable* (*pseudo-simultaneously flippable*) by Hoffmann et al. [18], where a lower bound of $\max {\{n/{2} - 2,h-3\}}$ is shown for such ps-flippable edges. The proof (not the statement) of [18, Lemma 1.3] implies Lemma 4.10 above. We want to claim that the proof given above, including the proof of the Unoriented Edges Lemma 4.9 employed, is more concise. We emphasize, though, that our proof of the Unoriented Edges Lemma and its set-up is clearly inspired by the proof of the $\lceil {n}/{2}-2 \rceil$-bound for flippable edges in [20] (see also [25] for Lemma 4.9 (i)).

We conclude this section by mentioning a few further applications of Lemma 4.9 (which will play no role in the rest of the paper). A set of pairwise independently flippable edges is often called *simultaneously flippable* in the literature. Here is a streamlined proof of the known tight lower bound on their number by Souvaine et al. from 2011 [35], based on the Unoriented Edges Lemma 4.9.

#### Theorem 4.12

[35] Every triangulation *T* of *P* has a set of at least $\lceil ({n-4})/{5}\rceil$ edges that are pairwise independently flippable (simultaneously flippable).

#### Proof

Let *S* be a $\preceq$-maximal coarsening of *T*. Orient edges in *S* (all locked) to a locking endpoint (ties broken arbitrarily). Lemma 4.9(ii) guarantees at least $({n-4-({{\,\mathrm{\textsf {sl}\mathrm }\,}}{S}+\textsf {sl} ^*S)})/{2}$ unoriented edges in $\vec {S}$. Since all inner edges are oriented, ${{\,\mathrm{\textsf {sl}\mathrm }\,}}{S}+\textsf {sl} ^*S \ge n-4$ holds.

For each region *r* of *S* of slack *d*, *T* induces a triangulation of this $(d+3)$-gon. Its *d* diagonals can be 3-colored with no triangle incident to two edges of the same color (start with an edge and spread the coloring along the dual tree of the triangulation of *r*). Each color class offers a set of simultaneously flippable edges, one of size at least $\lceil {d}/{3} \rceil$. It is easy to verify that $\lceil {d}/{3}\rceil \ge ({d + \lceil d/2\rceil })/{5}$ (check for $d=0,1,2$; for $d\ge 3$, $\lceil {d}/{3} \rceil \ge {d}/{3} \ge ({d + (d+1)/2})/{5}\ge ({d + \lceil d/2\rceil })/{5}$). We combine the simultaneously flippable edges collected for each region of *S*, which gives an overall set of at least $({{{\,\mathrm{\textsf {sl}\mathrm }\,}}{S}+\textsf {sl} ^*S})/{5} \ge ({n-4})/{5}$ simultaneously flippable edges. $\square$

While the lower bounds of $({n-4})/{2}$ for the number of flippable edges, and of $({n-4})/{5}$ for the size of a set of simultaneously flippable edges are tight (see [20] and [14], respectively), it is interesting to observe from the proofs that both bounds cannot be simultaneously attained for the same point set. That is, if the number of flippable edges is close to its lower bound then this forces the existence of a set of more than $\lceil ({n-4})/{5}\rceil$ simultaneously flippable edges, and vice versa. This can be quantified as follows.

#### Theorem 4.13

If the number of flippable edges in a triangulation *T* of *P* is $\alpha$, and if the largest set of simultaneously flippable edges in *T* has size $\beta$, then $\alpha + \beta \ge {4(n-4)}/{5}$.

(The claim is still true if $\alpha$ is the largest size of a set of pseudo-simultaneously flippable edges, see [18].)

#### Proof

Let *S* be a $\preceq$-maximal coarsening of *T*, with $d_1,d_2,\ldots ,d_{|{\textsf {R} }S|}$ the slacks of the regions of *S*. Then $\alpha \ge \sum _i d_i$ and, by the proof of Theorem 4.12, $\beta \ge \sum _i \lceil {d_i}/{3}\rceil$. Note that $d + \lceil {d}/{3} \rceil \ge {4}(d + \lceil d/2\rceil )/5$ for all $d \in \mathbb {N}_0$ (for $d=0,1,2$ we have $d + \lceil {d}/{3} \rceil = d + \lceil d/2\rceil$ and for $d \ge 3$, $d + \lceil {d}/{3} \rceil \ge {4d}/{3} \ge {4(d + (d+1)/2)}/{5} \ge {4(d + \lceil d/2\rceil )}/{5}$). Therefore, $\alpha + \beta \ge {4({{\,\mathrm{\textsf {sl}\mathrm }\,}}{S} + \textsf {sl} ^*S)}/{5} \ge {4(n-4)}/{5}$ (Lemma 4.9 (ii)). $\square$

Let us conclude this section with an observation about interior-disjoint *k*-holes, as it emerged from a discussion with Manfred  Scheucher (see also [34]). A *k-hole* of *P* is a subset of *k* points in convex position whose convex hull is disjoint from all other points in *P*. A *k*-hole and an $\ell$-hole are called interior-disjoint, if their respective convex hulls are interior-disjoint (they can share up to two points). Harborth showed in 1978 [17], that every set of at least ten points has a 5-hole. This was recently strengthened to the existence of another interior-disjoint 4-hole [19, Theorem 2]. We show, how our framework allows an easy proof of this fact.

#### Theorem 4.14

[19] Every set *P* of at least ten points has a 5-hole and a 4-hole which are interior-disjoint.

#### Proof

Since *P* has a 5-hole [17], there is a subdivision of *P* with a region of slack 2. Consider a $\preceq$-maximal coarsening *S* of such a subdivision with *r* a region of largest slack. We know that ${{\,\mathrm{\textsf {sl}\mathrm }\,}}{r} \ge 2$. If ${{\,\mathrm{\textsf {sl}\mathrm }\,}}{r} \ge 4$, i.e., it is a *k*-gon with $k\ge 7$, then we can add a diagonal to *r* which divides *r* into a 5-gon and a $(k-3)$-gon which readily gives the claimed interior-disjoint holes. Otherwise, if $k \le 6$, we have ${{\,\mathrm{\textsf {sl}\mathrm }\,}}{r} +\textsf {sl} ^*r \le 5$, while ${{\,\mathrm{\textsf {sl}\mathrm }\,}}{S} + \textsf {sl} ^*S \ge n-4 \ge 6$ (Lemma 4.9 (ii)). Hence, there must be another region $r'\in {\textsf {R} }S$ with positive slack, i.e., a $k'$-gon with $k'\ge 4$. $\square$

## $\max {\{\lceil {n}/{2}-2\rceil , h-3\}}$-Bound for Full Triangulations

The proof of Theorem 1.7(ii) needs one more ingredient.

### Link of a Full Triangulation

The *link* of a triangulation is the graph representing the compatibility relation among its flippable edges. In Sect. 1.7, the intuition for links as counterparts of vertex figures in polytopes was briefly explained.

#### Definition 5.1

(*link of full triangulation*). For $T \in {{\mathcal {T}}}_{\textsf {full} }(P)$, the *link of* *T*, denoted ${\textsf {Lk} }_{\textsf {full} }T$, is the edge-weighted graph with vertices ${\textsf {F} }T:= \{e \in {\textsf {E} ^\circ }T \mid e \ \text {flippable in}\ T\}$ and edge set $\bigl \{\{e,f\}\in { {\textsf {F} }T \atopwithdelims ()2} \mid e,f~\text {compatible}\bigr \}$. The *weight of* an edge $\{e,f\}$ is 2 if *e* and *f* are independently flippable, and 3 if *e* and *f* are weakly independently flippable.

**Fig. 12:**
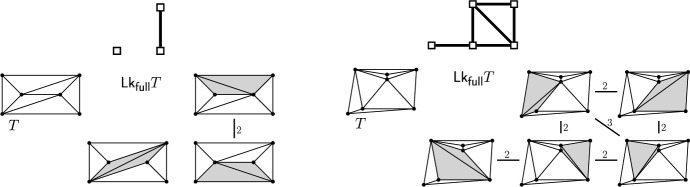
Two examples of links, the left link has three vertices, one of which is isolated. We indicate vertices of the link, i.e., $e \in {\textsf {F} }T$, as $T[e]=T[\nicefrac {e}{\overline{e}}]$ with $\textsf {terr} _{T}e=\textsf {terr} _{T[e]}\overline{e}$ shaded. Small examples are not typical: For large sets, the link is a dense highly connected graph

We will see that for proving Theorem 1.7 (ii) (in Sect. 5.2 below) it is enough to prove $\lceil {n}/{2} - 3 \rceil$-vertex connectivity of links. Here is the special property of links that will immediately show that the vertex connectivity is determined by the minimum vertex degree (via Lemma 2.4).

#### Lemma 5.2

For $$T \in {{\mathcal {T}}}_{\textsf {full} }(P)$$, the complement of $${\textsf {Lk} }_{\textsf {full} }T$$ has no cycle of length $$4$$, i.e., if $$(e_0, e_1, e_2, e_3)$$ are flippable edges in *T*, then there exists $$i\in \{0,1,2,3\}$$ such that $$e_i $$ is compatible with $$e_{i+1~mod ~4}$$.

#### Proof

**Fig. 13:**
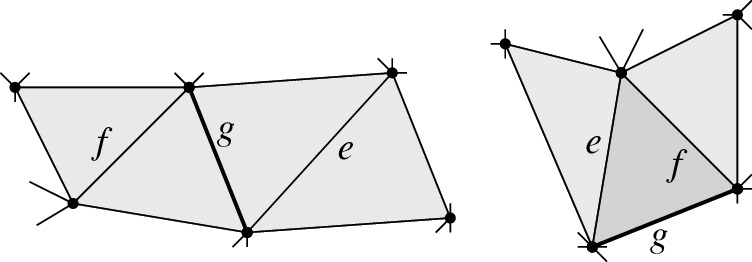
Edge *g* in the intersection of boundaries of territories of flippable edges *e* and *f*

For *e* and *f* flippable edges in *T* we show that there is at most one flippable edge *g* that is compatible with neither *e* nor *f*; this implies the lemma. Such a *g* has to be an edge of both ${{\,\mathrm{\textsf {terr}\mathrm }\,}}{e}$ and ${{\,\mathrm{\textsf {terr}\mathrm }\,}}{f}$. If ${{\,\mathrm{\textsf {terr}\mathrm }\,}}{e}$ and ${{\,\mathrm{\textsf {terr}\mathrm }\,}}{f}$ are disjoint, they share at most one edge, since ${{\,\mathrm{\textsf {terr}\mathrm }\,}}{e}$ and ${{\,\mathrm{\textsf {terr}\mathrm }\,}}{f}$ are convex, see Fig. 13, left. Otherwise, ${{\,\mathrm{\textsf {terr}\mathrm }\,}}{e}$ and ${{\,\mathrm{\textsf {terr}\mathrm }\,}}{f}$ overlap in a triangle $\Delta$, of which *e* and *f* are edges; the third edge *g* of this triangle is a common edge of ${{\,\mathrm{\textsf {terr}\mathrm }\,}}{e}$ and ${{\,\mathrm{\textsf {terr}\mathrm }\,}}{f}$, see Fig. 13, right. No other edge of ${{\,\mathrm{\textsf {terr}\mathrm }\,}}{f}$ can appear on the boundary of ${{\,\mathrm{\textsf {terr}\mathrm }\,}}{e}$: Consider the line $\lambda$ through edge *f*. All edges of ${{\,\mathrm{\textsf {terr}\mathrm }\,}}{f}$ other than *e* and *g* lie on the side of $\lambda$ opposite to $\Delta$, and ${{\,\mathrm{\textsf {terr}\mathrm }\,}}{e}$ is on the same side of $\lambda$ as $\Delta$, since it contains $\Delta$ and *f* is an edge of ${{\,\mathrm{\textsf {terr}\mathrm }\,}}{e}$; again, convexity of ${{\,\mathrm{\textsf {terr}\mathrm }\,}}{e}$ and of ${{\,\mathrm{\textsf {terr}\mathrm }\,}}{f}$ is essential here. $\square$

#### Lemma 5.3

For $T \in {{\mathcal {T}}}_{\textsf {full} }(P)$, the link ${\textsf {Lk} }_{\textsf {full} }T$ is $\max {\{\lceil {n}/{2}-3\rceil , h-4\}}$-vertex connected.

#### Proof

Every flippable edge in *T* is compatible with at least $\max {\{\lceil {n}/{2}-3\rceil , h-4\}}$ edges (Corollary 4.11 (ii)), i.e., the minimum vertex degree in ${\textsf {Lk} }_{\textsf {full} }T$ is at least $\max {\{\lceil {n}/{2}-3\rceil , h-4\}}$. ${\textsf {Lk} }_{\textsf {full} }T$ has no cycle of length $4$ in its complement (Lemma 5.2). The lemma follows (Lemma 2.4). $\square$

#### Lemma 5.4

Given flippable edges *e* and *f*, $e \ne f$, in $T \in {{\mathcal {T}}}_{\textsf {full} }(P)$, every *e*-*f* path of weight *w* in ${\textsf {Lk} }_{\textsf {full} }T$ induces a *T*-avoiding *T*[*e*]-*T*[*f*] path of length *w* in the edge flip graph, in a way that vertex-disjoint *e*-*f* paths in the link induce vertex-disjoint *T*[*e*]-*T*[*f*] paths.

**Fig. 14:**
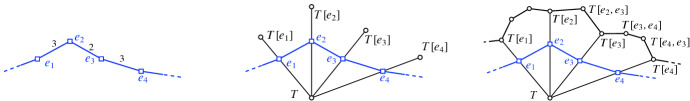
From a path in the link ${\textsf {Lk} }_{\textsf {full} }T$ to a path in the edge flip graph

#### Proof

Given an *e*-*f* path in ${\textsf {Lk} }_{\textsf {full} }T$, we replace each edge $\{z',z''\}$ on this path (i.e., $z'$ and $z''$ are compatible) by the path $(T[z'],T[z',z''],T[z''])$ or $(T[z'],T[z',z''],T[z'',z'],T[z''])$, depending on whether $z'$ and $z''$ are independently flippable (weight of $\{z',z''\}$ is $2$) or weakly independently flippable (weight of $\{z',z''\}$ is $3$), respectively, see Lemma 3.7 (Fig. 14). Note that the vertices $T[z',z'']$ and $T[z'',z']$ at distance $2$ from *T* (recall triangle-freeness, Corollary 3.4) on these substitutes satisfy $\textsf {E} T \setminus \textsf {E} T[z',z''] = \textsf {E} T \setminus \textsf {E} T[z'',z'] = \{z',z''\}$ ((2) in Lemma 3.3). Therefore, these vertices cannot appear on any substitute for another edge on the given *e*-*f* path, nor on substitutes for any other vertex-disjoint *e*-*f* path. Clearly, also the internal vertices at distance $1$ from *T* (i.e., of the form *T*[*z*]) are distinct from internal vertices at other vertex-disjoint *e*-*f* paths. And, obviously, we have not employed the vertex *T* for the substituting paths. $\square$

### Proof of Theorem 1.7 (ii)

#### Proof

We want to show that for $n \ge 5$, the edge flip graph is $\max {\{\lceil {n}/{2}-2\rceil , h-3\}}$-vertex connected. We employ the Local Menger Lemma 2.3. We know that the edge flip graph is connected [21]. What is left to show is that for any triangulation $T\in {{\mathcal {T}}}_{\textsf {full} }(P)$ and edges *e* and *f* flippable in *T*, at least $\max {\{\lceil {n}/{2}-2\rceil , h-3\}}$ vertex-disjoint *T*[*e*]-*T*[*f*] paths exist in the edge flip graph. ${\textsf {Lk} }_{\textsf {full} }T$ has at least $\max {\{\lceil {n}/{2}-3\rceil , h-4\}}$ vertex-disjoint *e*-*f* paths (Lemma 5.3 and Menger’s Theorem 2.2). Therefore, there are at least $\max {\{\lceil {n}/{2}-3\rceil , h-4\}}$ *T*-avoiding vertex-disjoint *T*[*e*]-*T*[*f*] paths (Lemma 5.4). The extra path (*T*[*e*], *T*, *T*[*f*]) yields the theorem. $\square$

## Covering the Edge Flip Graph with Polytopes

Recall from Sect. 1.3 that for a convex *k*-gon there is a $(k-3)$-polytope whose 1-skeleton is isomorphic to the edge flip graph of triangulations of the *k*-gon, an associahedron, which we denote by ${{\mathcal {A}}}_{k-3}$ (the index reflecting its dimension; to be precise, ${{\mathcal {A}}}_{k-3}$ is some representative realization of the associadedron); ${{\mathcal {A}}}_1$ is an edge, ${{\mathcal {A}}}_2$ is a convex pentagon, etc. If we consider all triangulations of the *k*-gon with a given diagonal present, we get a facet of ${{\mathcal {A}}}_{k-3}$; all facets of ${{\mathcal {A}}}_{k-3}$ can be obtained in this way. In general, the *d*-faces of ${{\mathcal {A}}}_{k-3}$ represent the triangulations where a certain set of $k-3-d$ diagonals is present, i.e., a subdivision. Suppose now that we have a subdivision *S* of *P* with nontriangular regions $r_1,r_2,\ldots ,r_m$, with $r_i$ of slack $d_i$. Then the set, ${{\mathcal {T}}}_{\textsf {full} }{\langle S \rangle }$, triangulations refining *S*, with its edge flip graph is represented by the product (see [39])

$$\begin{aligned} {{\mathcal {A}}}_{d_1,d_2,\ldots ,d_m} := {{\mathcal {A}}}_{d_1} \times {{\mathcal {A}}}_{d_2} \times \cdots \times {{\mathcal {A}}}_{d_m}, \end{aligned}$$

a *d*-dimensional polytope for $d:=d_1+d_2+\cdots +d_m = {{\,\mathrm{\textsf {sl}\mathrm }\,}}{S}$.

**Fig. 15:**
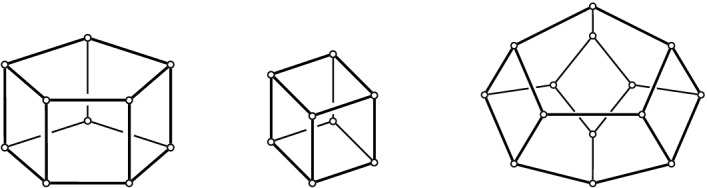
3-Dimensional products of associahedra for subdivisions of slack $3$: ${{\mathcal {A}}}_{1,2}$ (a pentagonal prism), ${{\mathcal {A}}}_{1,1,1}$ (a cube), and ${{\mathcal {A}}}_3$

It is now easy to see that every edge of the edge flip graph finds itself in the 1-skeleton of such a $\max {\{\lceil {n}/{2}-2\rceil , h-3\}}$-dimensional polytope contained in the edge flip graph (see also the discussion in Sect. 1.5).

### Theorem 6.1

For every edge $\{T,T[e]\}$ of the edge flip graph  there is an induced subgraph of the edge flip graph which contains the edge $\{T,T[e]\}$ and is isomorphic to the 1-skeleton of a product ${{\mathcal {A}}}_{d_1,d_2,\ldots ,d_m}$ of associahedra, where $d := d_1+d_2+\cdots +d_m \ge \max {\{\lceil {n}/{2}-2\rceil , h-3\}}$. Therefore, the edge flip graph (*vertices and edges*) *can be covered by* 1-*skeletons of* $\max {\{\lceil {n}/{2}-2\rceil , h-3\}}$-*dimensional products of associahedra contained in the edge flip graph*.

### Proof

Let *S* a $\preceq$-maximal coarsening of the subdivision $T_{-e}$ (*T* with *e* removed). Then ${{\,\mathrm{\textsf {sl}\mathrm }\,}}{S}\ge \max {\{\lceil {n}/{2}-2\rceil , h-3\}}$ holds (Coarsening Lemma 4.10). The subgraph induced by ${{\mathcal {T}}}_{\textsf {full} }{\langle S \rangle }$ gives the claimed product of associahedra. $\square$

One can strengthen this and show that any pair of incident edges in the edge flip graph is part of a subgraph isomorphic to the 1-skeleton of some $\lceil {n}/{2}-2\rceil$-dimensional polytope (a gluing of products of associahedra). This has been discussed in [37], but we decided to skip that part in this version.

## Partial Subdivisions—Slack and Order

In Sects. 7–9 we move on to proving Theorem 1.9, the $(n-3)$-vertex connectivity for the bistellar flip graph of partial triangulations. As indicated in the introduction, the proof will follow a similar line as for the edge flip graph, using subdivisions and links, but several new aspects and challenges will appear.

### Convention

From now on, in Sects. 7–11, we will mostly use ***triangulation*** short for “partial triangulation,” but we return to using “full triangulation” and “full subdivision.”

We define *partial subdivisions*, which form a poset in which the triangulations of *P* are the minimal elements—our definition is a specialization, to the plane and general position, of the established notion of a polyhedral subdivision [24]. These partial subdivisions are plane graphs, possibly with isolated points. Hence, it may be useful to point out a subtlety in the definition of regions of a plane graph (Definition 1.2): We defined them as the bounded connected components in the complement of the union the edges (as line segments in $\mathbb {R}^2$), not taking the isolated points into account. That is, regions can contain isolated points of the graph, and isolated points will not keep them from being convex.

### Definition 7.1

(*partial subdivision*). A *partial subdivision* *S* of *P* is a graph with ${\textsf {V} }S \subseteq P$ and ${\textsf {E} _\textsf {hull} }(P) \subseteq \textsf {E} S$ (hence ${{\,\mathrm{\textsf {xtr}\mathrm }\,}}{P} \subseteq {\textsf {V} }S$), and with all of its regions convex. Similar to triangulations (Definition 1.3), we define ${\textsf {E} ^\circ }S := \textsf {E} S \setminus {\textsf {E} _\textsf {hull} }$ (the *inner edges* of *S*) and ${\textsf {V} ^\circ }S: = {\textsf {V} }S \cap P^\circ$ (the *inner points* of *S*). Moreover, we let V$^{\textsf {inv}}{S}$ be the points in ${\textsf {V} }S$ which are isolated in *S*, the *bystanders* of *S*, and we let ${\textsf {V} }^{\textsf {inv}}{S} := {\textsf {V} ^\circ }S \setminus {\textsf {V} }^{\textsf {by}}{S}$, the *involved points* of *S*. For a region *r* of *S*, let ${\textsf {V} }r := \overline{r} \cap {\textsf {V} }S$ ($\overline{r}$ the closure of *r*), i.e., these are the vertices of the convex polygon *r* and the bystanders in this region. $\textsf {S} _{\textsf {triv} }= \textsf {S} _{\textsf {triv} }(P) := (P,{\textsf {E} _\textsf {hull} })$ is called the *trivial subdivision of* *P*.

Observe that a partial subdivision *S* is a full subdivision of *P* iff ${\textsf {V} }S = P$ and ${\textsf {V} }^{\textsf {by}}{S} = \emptyset$. Also, a partial subdivision *S* is a full subdivision of ${\textsf {V} }S \setminus {\textsf {V} }^{\textsf {by}}{S}$.

**Fig. 16:**
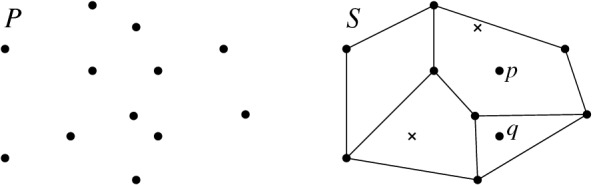
A set *P* and a partial subdivision *S* of *P*. Crosses indicate skipped points. Points *p* and *q* are bystanders. The region containing *p* has slack $6-3=3$ (Definition 7.2 below)

### Convention

From now on, in Sects. 7–11, we will mostly use ***subdivision*** for “partial subdivision.”

As it should have become clear by now, ${\textsf {V} }S$ is essential in the definition of a subdivision *S*, it is *not* simply the set of endpoints of edges in *S*, there are also bystanders. For example, for $T \in {{\mathcal {T}}}_{\textsf {part} }(P)$, all graphs $(P',\textsf {E} T)$ with ${\textsf {V} }T\subseteq P'\subseteq P$ are subdivisions of *P*, all different. ${\textsf {V} }S$ partitions into boundary points, involved points, and bystanders, i.e., ${\textsf {V} }S={{\,\mathrm{\textsf {xtr}\mathrm }\,}}{P}~\dot{\cup }~ {\textsf {V} }^{\textsf {inv}}{S} ~\dot{\cup }~ {\textsf {V} }^{\textsf {by}}{S}$. Moreover, there are the skipped points, $P\setminus {\textsf {V} }S=P^\circ \setminus {\textsf {V} ^\circ }S$.

A first important example of a subdivision is obtained from a triangulation *T* and an element *x* flippable in *T*, i.e., $\{T,T[x]\}$ is an edge of the bistellar flip graph:

$$\begin{aligned} T_{\pm x} : = ({\textsf {V} }T \cup {\textsf {V} }T[x], \textsf {E} T \cap \textsf {E} T[x]). \end{aligned}$$

If $x=e$ is a flippable edge, then $T_{\pm e}$ has one convex quadrilateral region *Q*; all other regions are triangular. We obtain *T* and *T*[*e*] from $T_{\pm e}$ by adding one or the other of the two diagonals of *Q* to $T_{\pm e}$. If $x=p$ is a flippable point, then $T_{\pm p}$ is almost a triangulation, all regions are triangular, except that $p \in {\textsf {V} }T_{\pm p}$ is a bystander. We obtain *T* and *T*[*p*] by either removing this point from $T_{\pm p}$ or by adding the three edges from *p* to the points of the triangular region in which *p* lies. The subdivision $T_{\pm x}$ is close to a triangulation and, in a sense, represents the flip between *T* and *T*[*x*]. To formalize and generalize this we generalize the notion of slack from full to partial subdivisions.

### Definition 7.2

(*slack of partial subdivision*). Given a subdivision *S* of *P*, we call a region of *S* *active* if it is not triangular or if it contains at least one point in ${\textsf {V} }S$ (necessarily a bystander) in its interior. For $r\in {\textsf {R} }S$, the *slack*, ${{\,\mathrm{\textsf {sl}\mathrm }\,}}{r}$, of *r* is $|{\textsf {V} }r|-3$. The *slack of* *S*, ${{\,\mathrm{\textsf {sl}\mathrm }\,}}{S}$, is the sum of slacks of its regions.

Note that a region is active iff it has nonzero slack.

### Observation 7.3

For a subdivision *S* with *s* bystanders we have

$$\begin{aligned} {{\,\mathrm{\textsf {sl}\mathrm }\,}}{S} = 3(|{\textsf {V} }S|-s) - 3 - h- |\textsf {E} S| + s = 3|{\textsf {V} }S| - 3 - h- |\textsf {E} S| - 2s. \end{aligned}$$

### Proof

The slack of a region *r* equals the number of edges it takes to triangulate *r* (ignoring bystanders) plus the number of bystanders in *r*. Thus, ${{\,\mathrm{\textsf {sl}\mathrm }\,}}{S}$ is the number of edges it takes to triangulate $({\textsf {V} }S \setminus {\textsf {V} }^{\textsf {by}}{S}, \textsf {E} S)$ (a full subdivision of ${\textsf {V} }S \setminus {\textsf {V} }^{\textsf {by}}{S}$) plus $|{\textsf {V} }^{\textsf {by}}{S}|$. Now the claim follows from Lemma 4.6 (or Observation 4.7). $\square$

### Observation 7.4

Let *S* be a subdivision.

(i) ${{\,\mathrm{\textsf {sl}\mathrm }\,}}{S}=0$ iff *S* is a triangulation iff *S* has no active region.
(ii) ${{\,\mathrm{\textsf {sl}\mathrm }\,}}{S}=1$ iff *S* has exactly one active region of slack $1$; this region is either a convex quadrilateral, or a triangular region with one bystander in its interior.
(iii) ${{\,\mathrm{\textsf {sl}\mathrm }\,}}{S}=2$ iff *S* has either (a) exactly two active regions, both of slack $1$, or (b) exactly one active region of slack $2$, where this region is either a convex pentagon, or a convex quadrilateral with one bystander in its interior, or a triangular region with two bystanders in its interior.

**Fig. 17:**
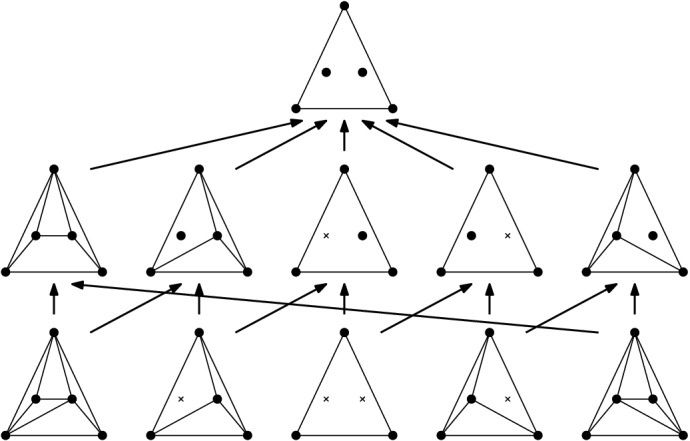
Hasse diagram of the partial order $\preceq$ for a set of five points

### Definition 7.5

(*coarsening, refinement*). For subdivisions $S_1$ and $S_2$ of *P*, $S_2$ *coarsens* $S_1$, in symbols $S_2 \succeq S_1$, if ${\textsf {V} }S_2 \supseteq {\textsf {V} }S_1$, and $\textsf {E} S_2 \subseteq \textsf {E} S_1$. We also say that $S_1$ *refines* $S_2$, ($S_1 \preceq S_2$).

The example in Fig. 17 hides some of the intricacies of the partial order $\preceq$; e.g., in general, it is not true that all paths from a triangulation to $\textsf {S} _{\textsf {triv} }$ have the same length $n-3$. $\textsf {S} _{\textsf {triv} }$ is the unique coarsest ($\preceq$-maximal) element (quite contrary to the poset of full subdivisions, where there were several $\preceq$-maximal full subdivisions). The triangulations (i.e., subdivisions of slack 0) are the minimal elements.

### Definition 7.6

(*set of refining partial triangulations*). For a subdivision *S* of *P* we let ${{\mathcal {T}}}_{\textsf {part} }{\langle S \rangle } := \{ T \in {{\mathcal {T}}}_{\textsf {part} }(P)\mid T \preceq S\}$.

Note that ${{\mathcal {T}}}_{\textsf {part} }{\langle \textsf {S} _{\textsf {triv} } \rangle } = {{\mathcal {T}}}_{\textsf {part} }(P)$ and for *x* flippable in *T*, ${{\mathcal {T}}}_{\textsf {part} }{\langle T_{\pm x} \rangle } = \{T,T[x]\}$.

### Observation 7.7

**Fig. 18:**
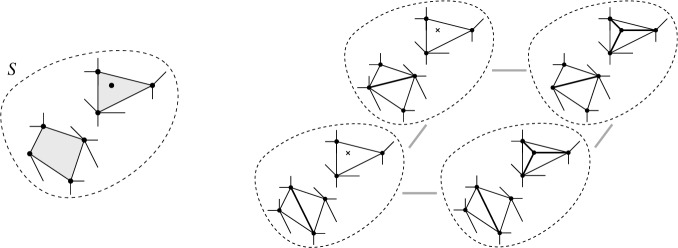
A subdivision *S* with two active regions of slack $1$ each. ${{\mathcal {T}}}_{\textsf {part} }{\langle S \rangle }$ spans a 4-cycle in the bistellar flip graph

(i) Any subdivision *S* of slack $1$ of *P* equals $T_{\pm x}$ for some triangulation $T \preceq S$ and some *x* flippable in *T*. (ii) Let *S* be a subdivision of slack $2$ of *P*. If there are exactly two active regions in *S* (of slack $1$ each), then ${{\mathcal {T}}}_{\textsf {part} }{\langle S \rangle }$ has cardinality $4$, spanning a 4-cycle in the bistellar flip graph of *P* (Fig. 18). If there is exactly one active region in *S* (of slack $2$), then ${{\mathcal {T}}}_{\textsf {part} }{\langle S \rangle }$ has cardinality $5$ , spanning a 5-cycle (see Fig. 2).

### Lemma 7.8

Any proper refinement *S* of a subdivision $S'$ of slack $2$ has slack at most $1$.

### Proof

For a refinement of $S'$ we add *m* edges, thereby involving $s'$ bystanders, and we remove $s''$ bystanders (some of these parameters may be 0, but not all, if the refinement is proper). We have ${{\,\mathrm{\textsf {sl}\mathrm }\,}}{S} = {{\,\mathrm{\textsf {sl}\mathrm }\,}}{S'} - (m - 2s' + s'')$ (easy consequence of Observation 7.3) and we want to show $m - 2s' + s''> 0$.

Since ${{\,\mathrm{\textsf {sl}\mathrm }\,}}{S'}=2$, $S'$ has at most two bystanders and thus $s'\le 2$. If $s'=0$, then $m-2s'+s''>0$ holds, since some of the three parameters have to be positive. If $s'=1$, we observe that we need at least three edges to involve a bystander and $m-2s'\ge 3 -2\cdot 1=1$. If $s'=2$, we need at least five edges to involve two bystanders and $m-2s' \ge 5 -2 \cdot 2=1$. $\square$

**Fig. 19:**
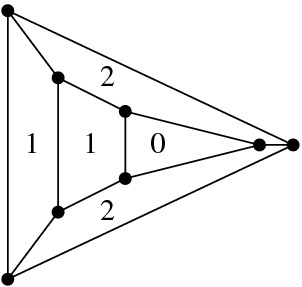
Eight points, with a subdivision of slack $6$, a refinement of $\textsf {S} _{\textsf {triv} }$ of slack $8-3 =5$

For $D \ge 3$, a proper refinement of a subdivision of slack *D* can have slack *D* or even higher (Fig. 19). The proof fails, since we can involve three bystanders with six edges. Intuitively, as briefly alluded to at the end of Sect. 1.3 (for the special case of convex position), one can think of the subdivisions as the faces of a higher-dimensional geometric structure behind the bistellar flip graph, with slack playing the role of dimension, analogous to the secondary polytope for regular triangulations. The following lemma shows that—for slack at most $2$—we have the property corresponding to the fact that faces of dimension *d* are either equal, or intersect in a common face of smaller dimension (possibly empty). This correspondence fails for slack exceeding 2.

### Lemma 7.9

(i) For subdivisions $S_1$ and $S_2$ of slack $2$, ${{\mathcal {T}}}_{\textsf {part} }{\langle S_1 \rangle } \cap {{\mathcal {T}}}_{\textsf {part} }{\langle S_2 \rangle }$ is either (a) empty, (b) equals $\{T\}$ for some triangulation *T*, (c) equals $\{T,T[x]\}$ for some triangulation *T* and some flippable element *x*, or (d) $S_1 = S_2$.
(ii) Let *x* and *y* be two distinct flippable elements in triangulation  *T*. If there is a subdivision *S* of slack $2$ with $\{T[x],T,T[y]\} \subseteq {{\mathcal {T}}}_{\textsf {part} }{\langle S \rangle }$, then this *S* is unique.

### Proof

If ${{\mathcal {T}}}_{\textsf {part} }{\langle S_1 \rangle } \cap {{\mathcal {T}}}_{\textsf {part} }{\langle S_2 \rangle }$ contains some triangulation, then we easily see that $S_1 \wedge S_2 := ({\textsf {V} }S_1 \cap {\textsf {V} }S_2, \textsf {E} S_1 \cup \textsf {E} S_2)$ is a subdivision, and ${{\mathcal {T}}}_{\textsf {part} }{\langle S_1 \wedge S_2 \rangle }={{\mathcal {T}}}_{\textsf {part} }{\langle S_1 \rangle } \cap {{\mathcal {T}}}_{\textsf {part} }{\langle S_2 \rangle }$.

(i) If (a) does not apply, let $S := S_1 \wedge S_2$, a subdivision with ${{\mathcal {T}}}_{\textsf {part} }{\langle S \rangle } = {{\mathcal {T}}}_{\textsf {part} }{\langle S_1 \rangle } \cap {{\mathcal {T}}}_{\textsf {part} }{\langle S_2 \rangle }$. If ${{\,\mathrm{\textsf {sl}\mathrm }\,}}{S}=0$ we have property (b), if ${{\,\mathrm{\textsf {sl}\mathrm }\,}}{S}=1$ we have property (c). In the remaining case ${{\,\mathrm{\textsf {sl}\mathrm }\,}}{S} \ge 2$, *S* is a refinement of $S_1$ and of $S_2$. Lemma 7.8 tells us that *S* cannot be a proper refinement of $S_1$, hence $S=S_1$; similarly, $S=S_2$, hence $S_1=S_2$.
(ii) Suppose $S_1$ and $S_2$ are subdivisions of slack $2$ with $\{T[x],T,T[y]\}\subseteq {{\mathcal {T}}}_{\textsf {part} }{\langle S_1 \rangle }\cap {{\mathcal {T}}}_{\textsf {part} }{\langle S_2 \rangle }$. Since options (a)–(c) above cannot apply, we are left with $S_1=S_2$.

$$\square $$

Two edges incident to a vertex of a polytope may span a 2-face, or not; same here, which gives rise to the following definition:

### Definition 7.10

(*compatible elements*). Two distinct flippable elements $x,y \in {\textsf {V} ^\circ }T \cup {\textsf {E} ^\circ }T$ are called *compatible in* *T*, in symbols $x\diamond y$, if there is a subdivision $T_{\pm x,y} \succeq T$ of slack $2$, s.t. $\{T[x],T,T[y]\}\subseteq {{\mathcal {T}}}_{\textsf {part} }{\langle T_{\pm x,y} \rangle }$. (Note that $T_{\pm x,y}$ is unique, by Lemma 7.9 (ii).) Otherwise, *x* and *y* are called *incompatible in* *T*, in symbols $x{\not \!\diamond \,\,}y$.

**Fig. 20:**
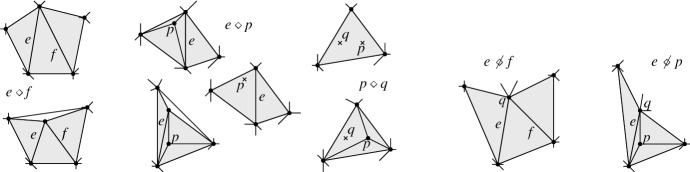
Compatible elements (with overlapping incident regions), all contained in a $5$-cycle, see Fig. 2, and incompatible elements (two rightmost, where *q* is assumed to have degree at least $5$). Shaded areas are unions of incident regions of flippable elements (not the active region in $T_{\pm x,y}$!)

This needs some time to digest. In particular, if two flippable edges *e* and *f* share a common endpoint of degree $4$, then they are compatible (Fig. 20, bottom left), quite contrary to the situation for full triangulations as treated in Sect. 3.3 (see Definition 3.6). The configurations of two flippable but incompatible elements are shown in Fig. 20 (two rightmost): (a) Two flippable edges *e* and *f* whose removal creates a nonconvex pentagon and whose common endpoint *q* has degree at least $5$. (b) A flippable edge *e* and a flippable point *p* of degree $3$ whose removal creates a nonconvex quadrilateral region whose reflex point *q* has degree at least $5$ in the triangulation. What is essential for us is that whenever *x* and *y* are compatible in a triangulation *T*, then there is a cycle of length $4$ or $5$ containing (*T*[*x*], *T*, *T*[*y*]), and therefore, apart from the path (*T*[*x*], *T*, *T*[*y*]), there exists a *T*-avoiding *T*[*x*]-*T*[*y*] path of length $2$ or $3$.

### Observation 7.11

Let $$T \in {{\mathcal {T}}}_{\textsf {part} }(P)$$.

(i) A skipped point $p \in P^\circ \setminus {\textsf {V} ^\circ }{T}$ is compatible with every flippable element of *T*.
(ii) Any two flippable points $p,q \in P^\circ$ are compatible.

## Coarsening Partial Subdivisions

**Fig. 21:**
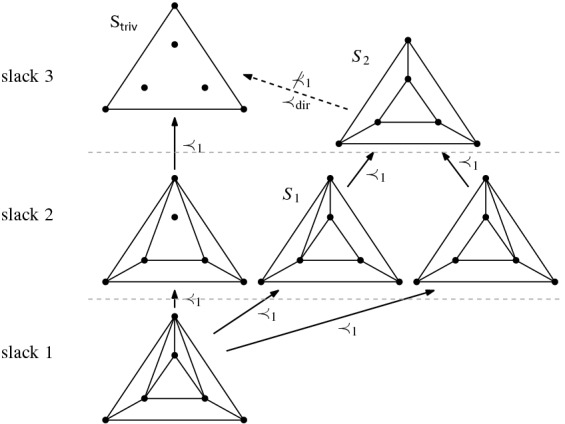
Relations $\prec _{\textrm{dir}}$ and $\prec _1$ on some subdivisions of a 6-point set. ${{\,\mathrm{\textsf {sl}\mathrm }\,}}{S_1} = 2$, ${{\,\mathrm{\textsf {sl}\mathrm }\,}}{S_2} = 3$, ${{\,\mathrm{\textsf {sl}\mathrm }\,}}{\textsf {S} _{\textsf {triv} }}=3$. Note that $S_2 \prec _{\textrm{dir}}\textsf {S} _{\textsf {triv} }$ but $S_2 \not \prec _1\textsf {S} _{\textsf {triv} }$, and that $S_1\preceq \textsf {S} _{\textsf {triv} }$ with ${{\,\mathrm{\textsf {sl}\mathrm }\,}}{\textsf {S} _{\textsf {triv} }} = {{\,\mathrm{\textsf {sl}\mathrm }\,}}{S_1} + 1$ but $S_1 \not \prec _1\textsf {S} _{\textsf {triv} }$, since $S_1 \not \prec _{\textrm{dir}}\textsf {S} _{\textsf {triv} }$

As in Sects. 4 and 5 for full triangulations, the existence of many coarsenings is essential for the vertex connectivity of the bistellar flip graph. However, note right away that going via $\preceq$-maximal subdivisions—as for full subdivisions—will not work: Here, for partial subdivisions, there is a unique $\preceq$-maximal element, the trivial subdivision. Moreover, note that for full subdivisions (as employed in Sect. 4), if $S_1 \preceq S_2$, then $(S_1,S_2)$ is an edge in the Hasse-diagram of the partial order $\preceq$ iff ${{\,\mathrm{\textsf {sl}\mathrm }\,}}{S_2} = {{\,\mathrm{\textsf {sl}\mathrm }\,}}{S_1}+1$. For partial subdivisions, this is not the case (Fig. 21).

### Definition 8.1

(*direct, perfect coarsening*). Let $S_1$ and $S_2$ be subdivisions.

(i) We call $S_2$ a *direct coarsening of* $S_1$ (and $S_1$ a *direct refinement of* $S_2$), in symbols $S_1 \prec _{\textrm{dir}}S_2$, if $S_1\preceq S_2$ and any subdivision *S* with $S_1 \preceq S \preceq S_2$ satisfies $S \in \{S_1,S_2\}$ (equivalently, if $(S_1,S_2)$ is an edge in the Hasse diagram of $\preceq$).
(ii) We call $S_2$ a *perfect coarsening of* $S_1$ ($S_1$ a *perfect refinement of* $S_2$), in symbols $S_1 \prec _1S_2$, if $S_1 \prec _{\textrm{dir}}S_2$ and ${{\,\mathrm{\textsf {sl}\mathrm }\,}}{S_2} = {{\,\mathrm{\textsf {sl}\mathrm }\,}}{S_1}+1$.
(iii) $\prec ^*_1$ is the reflexive transitive closure of $\prec _1$.

The reflexive transitive closure of $\prec _{\textrm{dir}}$ is exactly $\preceq$, while $\prec ^*_1\subseteq \preceq$ and, in general, the inclusion is proper.

**Fig. 22:**
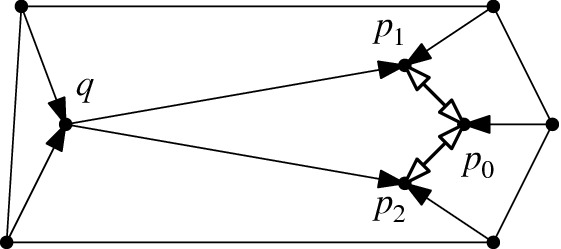
A subdivision, edges are oriented to endpoints where locked (not what we called a partial orientation in Definition 4.8, since some edges are doubly oriented). Removing the three edges incident to $p_0$ does not yield a subdivision, since a reflex angle occurs at $p_1$ and $p_2$. The edges incident to $\{p_0,p_1,p_2\}$ are not locked outside this set, but removing all incident edges creates a reflex angle at point *q*

To motivate the upcoming definitions, let us discuss a few possibilities of coarsenings, direct coarsenings and perfect coarsenings. There are the simple operations of removing an unlocked edge, and of adding a skipped point $p \in P \setminus {\textsf {V} }S$ as a bystander. For a triangulation, we can isolate a point of degree $3$. How does this generalize to subdivisions? Removing the edges incident to a point of degree $3$ does not work if some incident edge is locked at its other endpoint (e.g., $p_0$ in Fig. 22). If, however, no edge incident to a given point *p* (of any degree) is locked at the respective other endpoint, then we can isolate this point for a coarsening $S'$. Unless *p* has degree $3$, $S'$ is not a direct coarsening of *S*, though. If *p* has degree at least $4$, some incident edge, say *e*, is not locked at *p*, thus not locked at all, and therefore, $S \preceq S'' \preceq S'$ for $S'':= ({\textsf {V} }S,\textsf {E} S \setminus \{e\})$. Finally, suppose we want to isolate all points in a set *U* of points for obtaining a coarsening $S'$. For this to work, it is necessary that no edge *e* connecting *U* with the outside is locked at the endpoint of *e* not in *U*. However, this is not a sufficient condition, because several edges connecting *U* with a point not in *U* can collectively create a reflex vertex by their removal (e.g., $U=\{p_0,p_1,p_2\}$ in Fig. 22). Moreover, for $S \prec _{\textrm{dir}}S'$ to hold, *U* cannot be incident to unlocked edges, and no nonempty subset of *U* can be suitable for such an isolation operation.

### Definition 8.2

(*prime, perfect coarsener, increment*). Let *S* be a subdivision and let $\emptyset \ne U \subseteq {\textsf {V} ^\circ }S$.

(i) *U* is called a *coarsener*, if (a) every point in *U* is incident to at least one edge in *S* (i.e., $U \cap {\textsf {V} }^{\textsf {by}}{S} = \emptyset$), and (b) removal of the set $E_U$ of all edges incident to *U* in *S* yields a subdivision $S'$ where *U* is exactly the set of new bystanders, i.e., ${\textsf {V} }^{\textsf {by}}{S'} \setminus {\textsf {V} }^{\textsf {by}}{S} = U$.
(ii) If *U* is a coarsener, the *increment of* *U*, ${{\,\mathrm{\textsf {inc}\mathrm }\,}}{U}$, is defined as $|E_U| - 2|U|$.
(iii) *U* is called a *prime coarsener*, if (a) *U* is a coarsener, (b) *U* is a minimal coarsener, i.e., no proper subset of *U* is a coarsener, and (c) all edges incident to *U* are locked (i.e., removal of an individual edge incident to a point in *U* does not yield a subdivision).
(iv) *U* is called a *perfect coarsener*, if (a) *U* is a prime coarsener, and (b) ${{\,\mathrm{\textsf {inc}\mathrm }\,}}{U} = 1$.

**Fig. 23:**
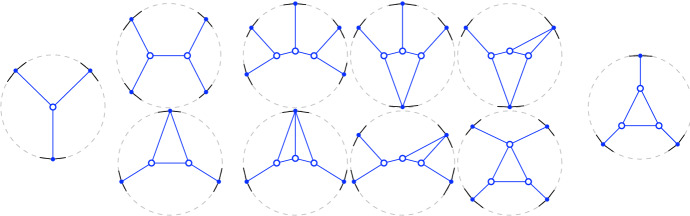
Prime coarseners, all perfect, except for the rightmost one (with $\textsf {inc} =6-2\cdot 3=0$, see Definition 8.2 (ii)). The points in a coarsener are indicated by hollow points. Removal of the edges incident to the points in a coarsener must not create reflex angles at the connecting points, here filled small points, to the outside. Otherwise, properties like coarsener, prime coarsener, and perfect coarsener depend only on the defining set and its incident edges and are independent of the rest of the graph

Examples of prime coarseners are given in Fig. 23.

### Remark 1

**Fig. 24:**
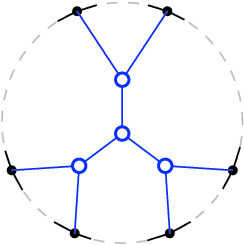
A prime coarsener of four points where there is a subset of three points which cover all edges to be removed in the associated coarsening step. The coarsener has increment $9-2\cdot 4 = 1$, hence it is perfect

Why is it necessary to put the extra condition “*where* *U* *is exactly the set of new bystanders, i.e.,* ${\textsf {V} }^{\textsf {by}}{S'} \setminus {\textsf {V} }^{\textsf {by}}{S} = U$” in Definition 8.2 (i)? *U* is supposed to be the set of points that get isolated in the coarsening step, this is essential for the definition of increment. However, there might be a subset of *U* that covers already all edges incident to *U* (see Fig. 24). We thank Valentin Stoppiello for pointing this out, it was overlooked by us in the earlier version [38].

The following observation, a simple consequence of Observation 7.3, explains the term “increment”. Let us note right away, that the increment can be negative.

### Observation 8.3

Let *S* be a subdivision with coarsener *U*, and let $S'$ be the subdivision obtained from *S* by removing all edges incident to *U*. Then ${{\,\mathrm{\textsf {sl}\mathrm }\,}}{S'}= {{\,\mathrm{\textsf {sl}\mathrm }\,}}{S} + {{\,\mathrm{\textsf {inc}\mathrm }\,}}{U}$.

### Observation 8.4

(i) Every subdivision *S* with ${\textsf {E} ^\circ }S\ne \emptyset$ has a coarsener (the set ).
(ii) If $U_1$ and $U_2$ are coarseners, then $U_1 \cap U_2$ is a coarsener, unless $U_1$ and $U_2$ are disjoint.
(iii) If $U_1$ and $U_2$ are prime coarseners, then $U_1=U_2$ or $U_1 \cap U_2 = \emptyset$.
(iv) If *U* is a prime coarsener, then the subgraph of *S* induced by *U* is connected.

The following observation lists all ways of obtaining direct and perfect coarsenings.

### Observation 8.5

Let $S=(V,E)$ and $S'$ be subdivisions.

(i) $S'$ is a direct coarsening of *S* iff it is obtained from *S* by one of the following.
  ***Adding a single point:*** For $p\in P \setminus V$, $S'=(V \cup \{p\},E)$ (with ${{\,\mathrm{\textsf {sl}\mathrm }\,}}{S'} = {{\,\mathrm{\textsf {sl}\mathrm }\,}}{S}+1$).
  ***Removing a single unlocked edge:*** For $e \in E$, not locked by either of its two endpoints, $S'=(V,E \setminus \{e\})$ (with ${{\,\mathrm{\textsf {sl}\mathrm }\,}}{S'} = {{\,\mathrm{\textsf {sl}\mathrm }\,}}{S}+1$).
  ***Isolating a prime coarsener:*** For *U* a prime coarsener in *S*, $S'$ is obtained from *S* by removal of the set, $E_U$, of all edges incident to points in *U*, i.e., $S'=(V,E \setminus E_U)$ (with ${{\,\mathrm{\textsf {sl}\mathrm }\,}}{S'} = {{\,\mathrm{\textsf {sl}\mathrm }\,}}{S} + {{\,\mathrm{\textsf {inc}\mathrm }\,}}{U}$).
(ii) $S'$ is a perfect coarsening of *S* iff it is obtained from *S* by adding a single point, removing a single unlocked edge, or by isolating a perfect coarsener.

We are prepared for the right formulation and proof of the Coarsening Lemma.

### Lemma 8.6

(Coarsening Lemma for partial subdivisions). Every subdivision of slack *D* has at least $n-3-D$ perfect coarsenings (i.e., direct coarsenings of slack $D+1$).

### Proof

We start with the case $D=0$, i.e., we have a triangulation *T* and we want to show that there are at least $n-3$ direct coarsenings of slack $1$. Let $N:= |{\textsf {V} }T|$. We orient inner locked edges to their locking endpoints (recall that in a triangulation there is at most one such endpoint for each inner edge). Let $C_i$, $i \in \mathbb {N}_0$, be the number of points $p\in {\textsf {V} ^\circ }T$ with indegree *i*. The number of unoriented, thus unlocked edges is at least $N-3-C_3$ (Lemma 4.9). There are $n -N$ subdivisions obtained from *T* by adding a single point, there are at least $N-3-C_3$ subdivisions obtained from *T* by removing a single unlocked edge, and there are $C_3$ direct coarsenings obtained from *T* by isolating an inner point of degree $3$. Adding up these numbers gives at least $n-3$ perfect coarsenings of *T*.

We let *S* be a subdivision of slack $D\ge 1$ assuming the assertion holds for slack less than *D*.

*Case 1*. There is a bystander $p_0 \in {\textsf {V} ^\circ }S$. Then $({\textsf {V} }S \setminus \{p_0\}, \textsf {E} S)$ is a subdivision of slack $D-1$ of $P \setminus \{p_0\}$ with at least $(n-1)-3-(D-1)=n-3-D$ perfect coarsenings of slack *D*. For each such perfect coarsening $S'$, the subdivision $({\textsf {V} }S' \cup \{p_0\}, \textsf {E} S')$ is a direct coarsening of *S* of slack $D+1$, thus a perfect coarsening.

**Fig. 25:**
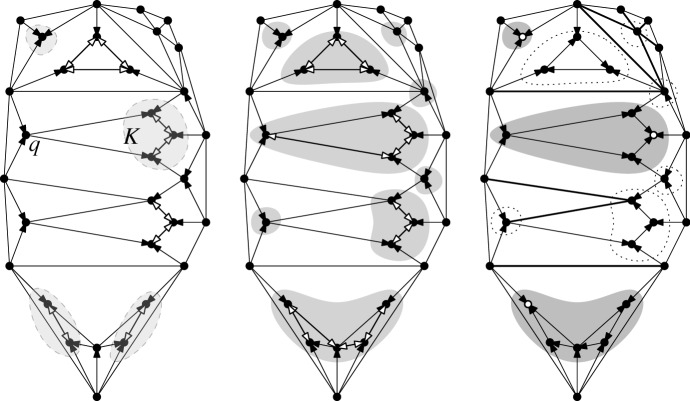
Orientation after phase 1, with candidate components shaded (left); after phase 2 (middle), with the connected components of $G^*$; after phase 3 (right), with unoriented edges bold (each of these can be removed for a coarsening of slack $1$ larger), and with the candidate components with a leader shaded (perfect coarseners)

*Case 2*. There is no bystander in *S*. Again we employ a partial orientation of *S*. The choice of the orientation is somewhat more intricate and we will proceed in three phases (Fig. 25). We keep the invariant that the unoriented inner edges are exactly the unlocked inner edges.

In a *first phase*, we orient all locked inner edges to *all* of their locking endpoints, i.e., we temporarily allow edges to be directed to both ends (to be corrected in the third phase); edges directed to both endpoints are called *mutual edges*. We can give the following interpretation to an edge directed from *p* to *q*: If we decide to isolate *p* (i.e., remove all incident edges of *p*) for a coarsening of *S*, then *q* becomes a reflex point of some region and we have to isolate *q* as well (i.e., every coarsener containing *p* must contain *q* as well). In particular, if $\{p,q\}$ is a mutual edge, then either both or none of the points *p* and *q* will be isolated. In fact, if we consider the graph *G* with ${\textsf {V} }G :={\textsf {V} ^\circ }S$ and $\textsf {E} G$ the mutual edges in the current orientation, then in any coarsening of *S* either all points in a connected component of *G* are isolated, or none.

A connected component *K* of *G* is called a *candidate component*, (a) if all edges connecting *K* with points outside are directed towards *K*, (b) no point in *K* is incident to an unoriented edge, (c) all points in *K* have indegree $3$, and (d) the mutual edges in *K* do not form any cycle (i.e., they have to form a spanning tree of *K*). It follows that if *K* has *k* points then the number of edges is $3k - (k-1) = 2k+1$. The term “candidate” refers to the fact that removing all edges incident to *K* *seems* like a direct coarsening step with incrementing the slack by $1$ (Observation 7.3); however, while individual edges connecting *K* to the rest of the graph are not locked at their endpoints outside *K*, some of these edges collectively may actually create a reflex vertex in this way (see *K* and *q* in Fig. 25, left). So *K* is only a candidate for a perfect coarsener.

We start the *second phase* of orienting edges further. In the spirit of our remarks about candidate components of *G*, suppose *q* is an inner point outside a candidate *K* of *G* (thus all edges connecting *q* to *K* are directed from *q* to *K*), such that removing the edges connecting *q* to *K* creates a reflex angle at *q*. Then we orient one (and only one) of the edges connecting *q* to *K*, say $\{p,q\}$, also to *q*, thereby making this edge mutual. (The reader might be worried that *q* now joins the candidate component while possibly not having indegree $3$ as required in a candidate component. However, this just means that the enlarged component is not a candidate component, i.e., we have lost a candidate component.) We call all the edges connecting *K* to *q*, except for $\{p,q\}$, the *witnesses of the extra new orientation of* $\{p,q\}$ *from* *p* *to* *q*. We successively proceed orienting edges, with the graph *G* of mutual edges evolving in this way and candidate components growing or disappearing. (The reader will correctly observe that our approach is very conservative towards prime coarseners, but by what we observed and by what will follow, since we are interested only in perfect coarseners, we can afford to leave alone connected components other than the candidate components.) The process will clearly stop at some point when the second phase is completed. We freeze *G* and denote it by $G^*$.

Before we start the third phase, let us make a few crucial observations:

(i) If *p*, *q* are inner points in the same connected component of $G^*$, then any coarsener contains both or none (i.e., if a connected component is a coarsener, then it is prime). This holds after phase 1, and whenever we expand a connected component, it is maintained.
(ii) During the second phase, an edge can be witness only once, and it is and will never be directed to the endpoint where it witnesses. Why? (a) Before it becomes a witness, it connects different connected components of *G*, after that it is and stays in a connected component of *G*. (b) Before it becomes a witness, it is not directed to the endpoint to which it witnesses an orientation, after that it is and stays in a connected component of *G* and can therefore not get an extra direction. (An unoriented edge can never get an orientation and it can never be a witness.)
(iii) If we remove, conceptually, for each incoming edge of a point *q* the witnesses (which direct away from *q*) for the orientation of this edge to *q*, then among remaining incident edges, all the incoming edges are locked at *q* (an incoming edge that was oriented already in the first phase to *q* has no witness). In particular, the indegree of *q* cannot exceed $3$, and if *q* is incident to some not ingoing edge which is not a witness for any edge incoming at *q*, then the indegree of *q* is at most $2$. (We might generate incoming edges to a point *q* that are not consecutive around *q*.)
(iv) If an unoriented edge *e* connects two points of the same connected component of $G^*$, then both endpoints have indegree at most $2$ (recall that this edge *e* cannot be a witness at its endpoints). If an edge *e* is directed from a connected component *K* of $G^*$ to a point outside *K*, then the tail of this edge *e* has indegree at most $2$ (recall that *e* cannot be a witness at all, since its endpoints are in different connected components of $G^*$).
(v) A candidate component *K* of $G^*$ is a perfect coarsener. It is a coarsener (otherwise, we would have expanded it further), it is a prime coarsener (see (i) above) and ${{\,\mathrm{\textsf {inc}\mathrm }\,}}{K} = 1$ (we have argued before that a candidate component increases the slack by exactly $1$).

The *third phase* will make sure that each mutual edge loses exactly one direction. Our goal is to have in every connected component *K* of $G^*$ at most one point with indegree $3$. To be more precise, only candidate components have exactly one point with indegree $3$, others don’t. Consider a connected component *K*.

- If the mutual edges form cycles in *K*, choose such a cycle *c* and keep for each edge on *c* one orientation so that we have a directed cycle, counterclockwise, say. All other mutual edges in *K* keep the direction in decreasing distance in $G^*$ to *c*, ties broken arbitrarily. This completed, no point in *K* has indegree $3$, since there is always a mutual edge incident that decreases the distance to *c* and the incoming direction of this edge will be removed.
- If *K* has points of indegree at most $2$, choose one such point *p* with indegree at most $2$, orient all mutual edges in *K* in decreasing distance in $G^*$ to *p*, ties broken arbitrarily. Again, this completed, no point in *K* will have indegree $3$.
- If none of the above applies, the mutual edges of *K* form a spanning tree and all points in *K* have indegree $3$. Moreover, all edges connecting *K* with points outside are directed towards *K* and no edge within *K* is unoriented (violation of these properties force a point of indegree at most $2$). So this is a candidate component. We choose an arbitrary point *p* in *K*, call it the *leader of* *K*, and for all mutual edges keep the orientation of decreasing distance in $G^*$ to *p* (ties cannot occur, mutual edges form a tree). Now the leader *p* is the only point of *K* with indegree $3$, all other points in *K* have indegree exactly $2$.

Phase 3 is completed. Let us denote the obtained partial orientation of *S* as ${\vec {S}}^{*}$. It has identified certain connected components of $G^*$ which have a leader of indegree $3$. In fact, every point of indegree $3$ after phase 3 is part of a perfect coarsener (probably of size $1$).

We can now describe a sufficient supply of perfect coarsenings of *S*. Let $N:= |{\textsf {V} }S|$ and let $C_3$ be the number of points of indegree $3$ in ${\vec {S}}^{*}$. We know that there are at least $N-3-D - C_3$ unoriented inner edges (Lemma 4.9).

(I) There are $n-N$ perfect coarsenings obtained by adding a single point $p \in P \setminus {\textsf {V} }S$.
(II) There are at least $N-3-D- C_3$ perfect coarsenings obtained by removing a single unoriented inner edge in ${\vec {S}}^{*}$.
(III) And there are $C_3$ perfect coarsenings obtained by isolating all points in a candidate component in $G^*$ (with a leader of indegree $3$).

In this way we have identified at least $n-3-D$ perfect coarsenings. $\square$

Here are two immediate implications which we will need later: The first in the vertex connectivity proof in Sect. 9 and the second for the result about covering of the bistellar flip graph by $(n-3)$-polytopes in Sect. 11.

### Corollary 8.7

Let $$T\in {{\mathcal {T}}}_{\textsf {part} }(P)$$.

(i) *T* has at least $n-3$ flippable elements.
(ii) For every *x* flippable in *T* there are at least $n-4$ elements compatible with *x*.

Part (i) of the corollary was proved, without general position assumption, in [25, Theorem 2.1].

### Corollary 8.8

For every subdivision $S'$ with ${{\,\mathrm{\textsf {sl}\mathrm }\,}}{S'} \le n-3$ there is a subdivision *S* with $S' \prec ^*_1S$ and ${{\,\mathrm{\textsf {sl}\mathrm }\,}}{S} = n-3$.

## $(n-3)$-Connectivity for Partial Triangulations

To complete the proof of Theorem 1.9, the $(n-3)$-vertex connectivity of the bistellar flip graph, we need again links, now for partial triangulations, which are graphs that represent the compatibility relation among flippable elements (Definition 7.10).

### Link of a Partial Triangulation

Recall that if *x* is a flippable element in a triangulation *T* then $T_{\pm x}$ denotes the subdivision with ${{\mathcal {T}}}_{\textsf {part} }{\langle T_{\pm x} \rangle } =\{T,T[x]\}$, and if *y* is compatible with *x*, denoted $x\diamond y$, then $T_{\pm x,y}$ denotes the unique coarsening of slack $2$ of *T* with $\{T[x],T,T[y]\} \subseteq {{\mathcal {T}}}_{\textsf {part} }{\langle T_{\pm x,y} \rangle }$ (Definition 7.10).

#### Definition 9.1

(*link of partial triangulation*). For $T \in {{\mathcal {T}}}_{\textsf {part} }(P)$, the *link of* *T*, denoted ${\textsf {Lk} }_{\textsf {part} }T$, is the edge-weighted graph with vertices ${\textsf {F} }T:= \{x \in {\textsf {V} ^\circ }T \cup {\textsf {E} ^\circ }T \mid x\ \text {flippable in}\ T\}$ and edge set $\bigl \{\{x,y\}\in { {\textsf {F} }T \atopwithdelims ()2} \mid x \diamond y\bigr \}$. The *weight of* an edge $\{x,y\}$ is $|{{\mathcal {T}}}_{\textsf {part} }{\langle T_{\pm x,y} \rangle }| - 2$ (which is 2 or 3).

**Fig. 26:**
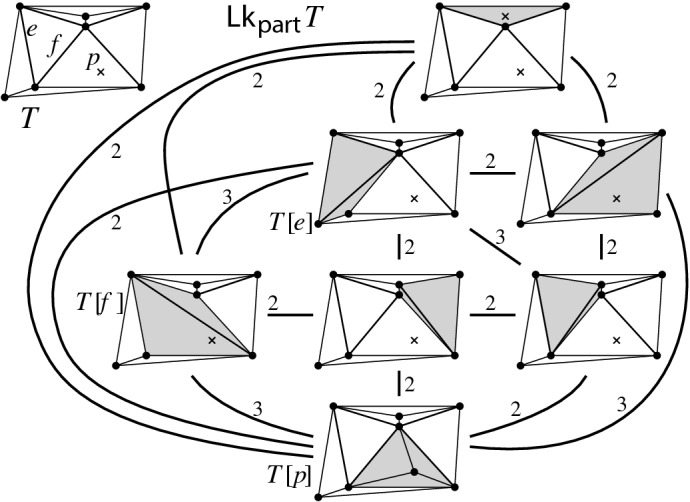
An example of a link of a partial triangulation *T*. Similar to Fig. 12, we indicate vertices $x\in {\textsf {F} }T$ of the link by the respective triangulations *T*[*x*]. Note that since *p* is skipped in *T*, it is compatible to all flippable elements of *T* (Observation 7.11 (i)), thus *p* connects to all vertices of the link. Observe that *e* and *f* are compatible, to be compared with the situation for full triangulations in Fig. 12

(See Sect. 1.7 for some intuition for this definition and Fig. 26 for an example.) We will see that it is enough to prove $(n-4)$-vertex connectivity of all links. The following lemma implies, via Lemma 2.4, that the vertex connectivity of links is determined by the minimum vertex degree.

#### Lemma 9.2

The complement of ${\textsf {Lk} }_{\textsf {part} }T$ has no cycle of length $4$, i.e., if $(x_0, x_1, x_2, x_3)$ are flippable elements in *T*, then there exists $i\in \{0,1,2,3\}$ such that $x_i \diamond x_{i+1~mod ~4}$.

#### Proof

Recall that all $p\in P^\circ \setminus {\textsf {V} ^\circ }T$ are flippable and compatible with every flippable element (Observation 7.11 (i)), so we can assume $\{x_0, x_1, x_2, x_3\} \subseteq {\textsf {V} ^\circ }T \cup {\textsf {E} ^\circ }T$. Moreover, if $p,q\in {\textsf {V} ^\circ }T$ are two distinct points flippable in *T*, then $p\diamond q$ (Observation 7.11 (ii)). Hence, we assume that no two consecutive elements in the cyclic sequence $(x_0,x_1,x_2,x_3)$ are points; w.l.o.g. let $x_0=e$ and $x_2=f$ be edges.

Recall from Definition 3.2, that for an inner edge *e* in a triangulation *T*, its *territory* ${{\,\mathrm{\textsf {terr}\mathrm }\,}}{e}$, is defined as the interior of the closure of the union of the two regions in *T* incident to *e*. Obviously, *e* is flippable in *T* iff the quadrilateral $\textsf {terr} _{T}e$ is convex. Note that for an element *x* to be incompatible with edge *e*, *x* must appear on the boundary of ${{\,\mathrm{\textsf {terr}\mathrm }\,}}{e}$, and analogously elements incompatible with *f* must appear on the boundary of ${{\,\mathrm{\textsf {terr}\mathrm }\,}}{f}$.

**Fig. 27:**
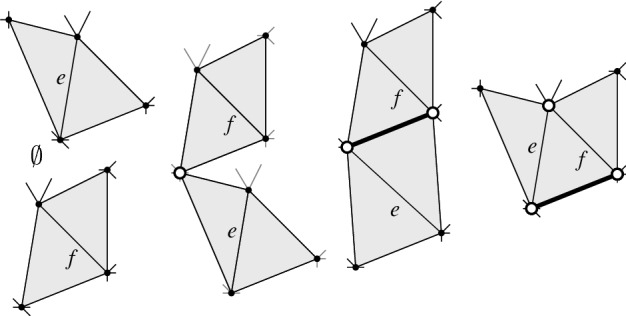
Intersections of boundaries of territories of two flippable edges

We show that there is at most one flippable element in the intersection of the boundaries of ${{\,\mathrm{\textsf {terr}\mathrm }\,}}{e}$ and ${{\,\mathrm{\textsf {terr}\mathrm }\,}}{f}$ (Fig. 27). This is obvious, if $\overline{{{\,\mathrm{\textsf {terr}\mathrm }\,}}{e}} \cap \overline{{{\,\mathrm{\textsf {terr}\mathrm }\,}}{f}}$ is empty or a single point (recall that $\overline{A}$ denotes the closure of $A \subseteq \mathbb {R}^2$). If this intersection is an edge and its two endpoints, we observe that among any edge and its two incident points, at most one element can be flippable (inner degree $3$ points cannot be adjacent and cannot be incident to a flippable edge). This covers already all possibilities if ${{\,\mathrm{\textsf {terr}\mathrm }\,}}{e}$ and ${{\,\mathrm{\textsf {terr}\mathrm }\,}}{f}$ are disjoint (since they are convex). Finally, $\overline{{{\,\mathrm{\textsf {terr}\mathrm }\,}}{e}} \cap \overline{{{\,\mathrm{\textsf {terr}\mathrm }\,}}{f}}$ can be a triangle (see argument in the proof of Lemma 5.2), in which case the common boundary consists of the common endpoint of *e* and *f*, clearly not flippable, and an edge with its two endpoints; again, at most one of these three can be flippable. $\square$

#### Lemma 9.3

Given a triangulation *T* with *x* and *y* flippable elements, $x \ne y$, every *x*-*y* path of weight *w* in ${\textsf {Lk} }_{\textsf {part} }T$ induces a *T*-avoiding *T*[*x*]-*T*[*y*] path of length *w* in the bistellar flip graph. Interior vertex-disjoint *x*-*y* paths in the link induce vertex-disjoint *T*[*x*]-*T*[*y*] paths.

**Fig. 28:**
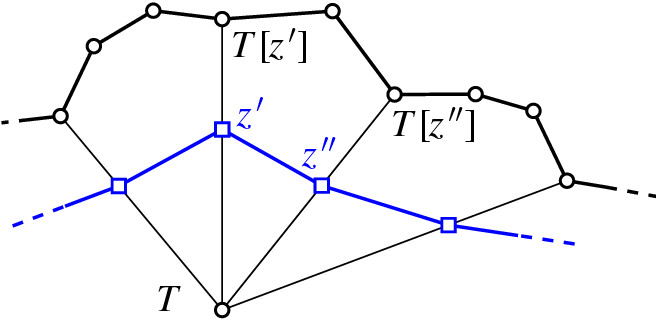
From a path in the link to a path in the bistellar flip graph

#### Proof

Given an *x*-*y* path, we replace every edge $\{z',z''\}$ on this path by $(T[z'],\ldots ,T[z''])$ (of length $2$ or $3$) which draws its ($1$ or $2$) internal vertices from ${{\mathcal {T}}}_{\textsf {part} }{\langle T_{\pm z',z''} \rangle } \setminus \{T[z'],T,T[z'']\}$ (Fig. 28); these vertices must have distance $2$ from *T* in the flip graph, while $T[z']$ and $T[z'']$ have distance $1$. In the resulting *T*[*x*]-*T*[*y*] path, all internal vertices adjacent to *T* (i.e., of the form *T*[*z*]) are distinct from internal vertices at other paths by assumption on the initial paths in the link. For vertices at distance $2$, suppose $T_1 \in {{\mathcal {T}}}_{\textsf {part} }{\langle T_{\pm z_1',z_1''} \rangle }$ coincides with $T_2 \in {{\mathcal {T}}}_{\textsf {part} }{\langle T_{\pm z_2',z_2''} \rangle }$, both at distance $2$ from *T*. Since ${{\,\mathrm{\textsf {sl}\mathrm }\,}}{T_{\pm z_1',z_1''}}={{\,\mathrm{\textsf {sl}\mathrm }\,}}{T_{\pm z_2',z_2''}}=2$, we have that ${{\mathcal {T}}}_{\textsf {part} }{\langle T_{\pm z_1',z_1''} \rangle } \cap {{\mathcal {T}}}_{\textsf {part} }{\langle T_{\pm z_2',z_2''} \rangle }$ either (a) equals $\{T\}$, (b) equals $\{T,T[z]\}$ for some *z*, or (c) $T_{\pm z_1',z_1''} = T_{\pm z_2',z_2''}$ (Lemma 7.9). In (a)–(b), $T_{\pm z_1',z_1''}$ and $T_{\pm z_2',z_2''}$ cannot possibly share a vertex at distance $2$ from *T*. Thus (c) holds. $T_{\pm z_1',z_1''} = T_{\pm z_2',z_2''}$ implies $\{z_1',z_1''\} = \{z_2',z_2''\}$. $\square$

#### Lemma 9.4

For $T \in {{\mathcal {T}}}_{\textsf {part} }(P)$, the link ${\textsf {Lk} }_{\textsf {part} }T$ is $(n-4)$-vertex connected.

#### Proof

Let *x* be a vertex of ${\textsf {Lk} }_{\textsf {part} }T$. $T_{\pm x}$, a subdivision of slack $1$, has at least $n-4$ perfect coarsenings of slack 2 (Lemma 8.6). Each such coarsening equals $T_{\pm x,y}$ for some $y \diamond x$, i.e., *y* is a neighbor of *x* in ${\textsf {Lk} }_{\textsf {part} }T$. Distinct coarsenings yield distinct compatible elements *y* (since $\{T[x],T,T[y]\} \subseteq {{\mathcal {T}}}_{\textsf {part} }{\langle T_{\pm x,y} \rangle }$ and ${{\mathcal {T}}}_{\textsf {part} }{\langle T_{\pm x,y} \rangle }$ spans a cycle, *T*[*y*] is determined as the other neighbor of *T* on this cycle). That is, the minimum vertex degree in ${\textsf {Lk} }_{\textsf {full} }T$ is at least $n-4$. ${\textsf {Lk} }_{\textsf {part} }T$ has no cycle of length $4$ in its complement (Lemma 9.2). The lemma follows by Lemma 2.4. $\square$

### Proof of Theorem 1.9

#### Proof

We know that the bistellar flip graph is connected [24, Sect. 3.4.1], and it has at least $n-2$ vertices, since it is nonempty and every vertex has degree at least $n-3$ (Corollary 8.7 (i)). Hence, for $(n-3)$-vertex connectivity, by the Local Menger Lemma 2.3 it is left to show that for any $T\in {{\mathcal {T}}}_{\textsf {part} }(P)$ and flippable elements *x* and *y*, there are at least $n-3$ vertex-disjoint *T*[*x*]-*T*[*y*] paths in the bistellar flip graph. Since ${\textsf {Lk} }_{\textsf {part} }T$ is $(n-4)$-vertex connected (Lemma 9.4), ${\textsf {Lk} }_{\textsf {part} }T$ has at least $n-4$ vertex-disjoint *x*-*y* paths (Menger’s Theorem 2.2). Therefore, there are at least $n-4$ vertex-disjoint *T*[*x*]-*T*[*y*] paths disjoint from *T* (Lemma 9.3). Together with the path (*T*[*x*], *T*, *T*[*y*]), the claim is established. $\square$

## Regular Subdivisions by Successive Perfect Refinements

**Fig. 29:**
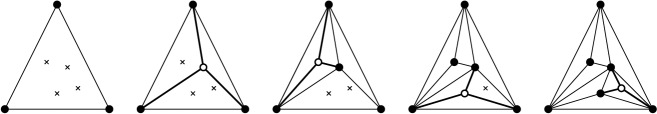
Stacked triangulations (which are always regular)

**Fig. 30:**
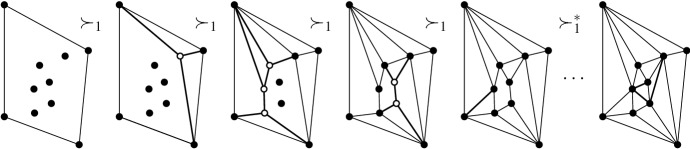
Successive perfect refinements of a trivial subdivision (all subdivisions regular)

Suppose $h=3$ and consider stacked triangulations of *P*, i.e., we start with the triangulation $({{\,\mathrm{\textsf {xtr}\mathrm }\,}}{P},{\textsf {E} _\textsf {hull} })$, and then we successively add points in $P^\circ$ by connecting a new point to the three vertices of the triangle where it lands in (Fig. 29). It is easily seen that this yields regular triangulations. The result of this section is the following sufficient condition for the regularity of a subdivision (Definition 10.2 below), which can be seen as a generalization of the regularity of stacked triangulations (Fig. 30). The condition is not necessary, see Sect. 10.2.

### Theorem 10.1

If $S \prec ^*_1\textsf {S} _{\textsf {triv} }$ for a subdivision *S*, then *S* is a regular subdivision.

In other words, all subdivisions, in particular, all triangulations in the $\prec _1$-lower closure of $\textsf {S} _{\textsf {triv} }$ are regular. This condition will eventually allow us to show the covering of the bistellar flip graph by graphs of $(n-3)$-polytopes. The proof of Theorem 10.1 stretches out over several definitions and lemmas with a conclusion in Sect. 10.6. Before we give a brief outline of this proof shortly in Sect. 10.3, we first introduce some notions.

### Height Functions, Liftings, and Regular Subdivisions

#### Definition 10.2

(*linear, compliant, realizing height function; regular subdivision*). A *height function on* $A \subseteq \mathbb {R}^2$ is a vector $\omega \in \mathbb {R}^A$, $p \mapsto \omega _p$. For $p = (x_p, y_p) \in A$, we let $p^{(\omega )}:=(x_p, y_p, \omega _p)$, and for $B \subseteq A$, we set $B^{(\omega )} := \{p^{(\omega )} ~|~ p \in B\}$. We say that $\omega$ is *linear on* $B \subseteq A$, if there exist *a*, *b*, and *c* in $\mathbb {R}$ such that $\omega _p = a x_p + b y_p + c$ for all $p \in B$, i.e., if $B^{(\omega )}$ is coplanar. Let *S* be a subdivision.

(i) A height function $\omega$ on ${\textsf {V} }S$ is *linear on* *S* if it is linear on ${\textsf {V} }S$. $\Lambda (S)$ denotes the set of linear height functions on *S* and for $A \subseteq {\textsf {V} }S$, $\Lambda _A(S)$ denotes the set of height functions on *S* linear on *A*.
(ii) A height function $\omega$ on ${\textsf {V} }S$ *complies with* *S*, (or is *S*-*compliant*), if for every region *r* of *S*, $\omega$ is linear on ${\textsf {V} }r$ (including bystanders). Let $\Gamma (S)$ be the set of *S*-compliant height functions.
(iii) A height function $\omega$ on ${\textsf {V} }S$ *realizes* *S*, if *S* is the projection of the lower convex hull of ${\textsf {V} }S^{(\omega )}$, with all points of ${\textsf {V} }S$ (also the bystanders) appearing on this lower convex hull.
(iv) *S* is called *regular* if there is a height function realizing *S*.

Compliant height functions constitute a relaxation of realizing height functions (and of linear height functions): Every realizing height function (and every linear height function) is compliant. All height functions on a triangulation *T* are compliant, i.e., $\Gamma (T) = \mathbb {R}^{{\textsf {V} }T}$. For the trivial subdivision $\textsf {S} _{\textsf {triv} }$, the compliant height functions are exactly the linear height functions, i.e., $\Gamma (\textsf {S} _{\textsf {triv} }) = \Lambda (\textsf {S} _{\textsf {triv} })$. While compliant height functions exist for all triangulations and the trivial subdivision, this is a non-trivial property for general subdivisions.

#### Lemma 10.3

Let *S* be a subdivision.

(i) $\Lambda (S)$ is a linear subspace of $\mathbb {R}^{{\textsf {V} }S}$ of dimension $\dim \Lambda (S) =3$. More generally, for every $B\subseteq {\textsf {V} }S$ with $|B|\ge 3$, $\Lambda _{B}(S)$ is a linear subspace of $\mathbb {R}^{{\textsf {V} }S}$ of dimension $|{\textsf {V} }S| - (|B| - 3)$.
(ii) $\Gamma (S) = \bigcap _{r \in {\textsf {R} }S} \Lambda _{{\textsf {V} }r}(S)$ (${\textsf {R} }S$ the set of regions of *S*).
(iii) $\Gamma (S)$ is a linear subspace of $\mathbb {R}^{{\textsf {V} }S}$ with $\Gamma (S) \supseteq \Lambda (S)$ and $\dim \Gamma (S)\ge |{\textsf {V} }S|-{{\,\mathrm{\textsf {sl}\mathrm }\,}}{S}$.

#### Proof

(i) is obvious and (ii) holds directly by definition. Now, with ${{\,\mathrm{\textsf {sl}\mathrm }\,}}{r} = |{\textsf {V} }r| - 3$ and ${{\,\mathrm{\textsf {sl}\mathrm }\,}}{S} = \sum _{r \in {\textsf {R} }S} (|{\textsf {V} }r| - 3)$, assertion (iii) is an immediate consequence of (i), (ii), and the fact that intersecting linear subspaces of co-dimension $d_1$ and $d_2$ yields a subspace of co-dimension at most $d_1+d_2$:

$$\begin{aligned} |{\textsf {V} }S| - \dim \Gamma (S){\mathop {\le }\limits ^{\mathrm{(ii)}}}\sum _{{r \in {\textsf {R} }S}}(|{\textsf {V} }S| - \dim \Lambda _{{\textsf {V} }r}(S)){\mathop {=}\limits ^{\mathrm{(i)}}}\sum _{{r \in {\textsf {R} }S}}(|{\textsf {V} }r| - 3) = {{\,\mathrm{\textsf {sl}\mathrm }\,}}{S}. \end{aligned}$$

$$\square $$

We see that if ${{\,\mathrm{\textsf {sl}\mathrm }\,}}{S} < |{\textsf {V} }S|-3$, there are always compliant height functions not in $\Lambda (S)$. In order to extract among those a realizing height function we consider mountains and valleys in the lifting given by a compliant height function.

#### Definition 10.4

($\omega$-*lifting*; $\omega$-*labeling*). Let $\omega \in \Gamma (S)$. The $\omega$-*lifting of* *S* is the unique piecewise linear function *f* on the convex hull of ${\textsf {V} }S$, that is linear on every region *r* of *S*, and . We call $e \in {\textsf {E} ^\circ }S$ a *mountain*, a *valley*, or *flat* in the $\omega$-lifting, depending on whether the directional derivative of function *f* decreases, increases, or remains constant, respectively, as one traverses the *f*-lifted edge from one side to the other (at a mountain, the function is locally strictly concave, at a valley it is locally strictly convex). The $\omega$-*labeling of* *S* assigns $\oplus$, $\ominus$, and 0 to each inner edge of *S*, depending on whether the lifted edge is a mountain, a valley, or flat, respectively.

**Fig. 31:**
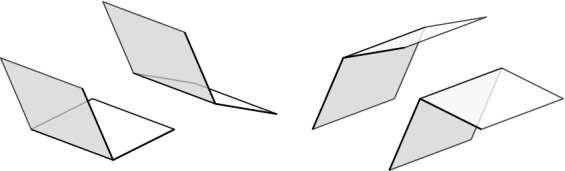
Valleys (left) and mountains (right)

#### Observation 10.5

Let $$\omega \in \Gamma (S)$$.

(i) $\omega$ is linear on *S* iff the $\omega$-labeling of *S* is constant 0.
(ii) $\omega$ realizes *S* iff the $\omega$-labeling of *S* is constant $\ominus$.

### Mother of Examples

**Fig. 32:**
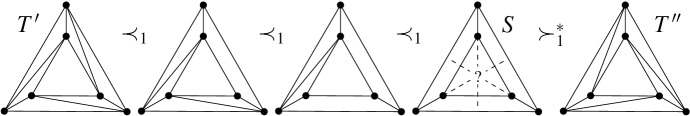
Exactly one of *S*, $T'$, and $T''$ is regular. Which one depends on how the dashed lines meet

In order to understand the subtleties of whether a subdivision is regular or not, we should briefly discuss the mother of examples, see [24]. For this consider the configuration in Fig. 32. Whether or not the displayed subdivisions are regular or not depends on how exactly the three dashed lines (as indicated in *S*) meet.

- If the three dashed lines meet in a common point, then *S* is a regular subdivision, but none of $T'$ and $T''$ is regular.
- If the three dashed lines do not meet in a common point, then *S* is not a regular subdivision, but one of $T'$ and $T''$ is regular, the other one not.

The example allows us to clarify a few points.

- The condition in Theorem 10.1 for regularity is not necessary (consider case (b) with $T'$ regular, and note $T'\not \prec ^*_1\textsf {S} _{\textsf {triv} }$). This is inherently so, since the condition in Theorem 10.1 depends only on the order type (or oriented matroid) of the point set. In fact, note that a perturbation of the point set does not change the order type of the set, but it affects how the dashed lines meet and, therefore, whether subdivisions are regular or not.
- The condition in Theorem 10.1*cannot* be generalized to: *If* $S \prec ^*_1S'$ *and* $S'$ *is regular, then* *S* *is regular.* In fact, adding a single edge in a subdivision may switch from regular to non-regular (case (a)). The right generalization will be given in the Regularity Preservation Lemma 10.10 below.

### Outline of Proof of Theorem 10.1

It is easy to see that if *p* is an inner point of degree $3$ in a triangulation  *T*, then for any height function $\omega$ (which, as we observed, is *T*-compliant), the $\omega$-labeling assigns the same value to the three edges incident to *p*. We will generalize this observation for an *S*-compliant height function $\omega$ in two ways:

(A) If $p \in {\textsf {V} ^\circ }S$ and not all incident edges are 0-labeled, then the $\oplus$-labeled and $\ominus$-labeled edges incident to *p* cannot be separated by a line through *p* (Lemma 10.7). (In particular, this forces the $\omega$-labeling to be constant on the edges incident to an inner point of degree $3$ in any subdivision.)
(B) If *K* is a perfect coarsener of *S*, then the $\omega$-labeling assigns the same label to all the edges $E_K$ incident to a perfect coarsener *K* (Lemma 10.9).

Here is another simple observation about an inner point *p* of degree $3$ in a triangulation  *T*. Removing the three edges incident to *p* (while keeping *p* as a bystander) yields a subdivision *S* with $T\prec _1S$. Obviously, if *S* is a regular subdivision, then *T* is a regular triangulation (this was behind our observation about stacked triangulations at the beginning of this section): Given a height function $\omega$ realizing *S*, we can always perturb *p* downwards (decrease $\omega _p$ by a sufficiently small value $\varepsilon$), obtaining a height function that realizes *T*. Again, this allows an appropriate generalization:

(C) Suppose *S* is a regular subdivision with $\dim \Gamma (S) = |{\textsf {V} }S| - {{\,\mathrm{\textsf {sl}\mathrm }\,}}{S}$. Then every perfect refinement $S'$ of *S*, i.e., $S' \prec _1S$, is regular, and, moreover, $\dim \Gamma (S') = |{\textsf {V} }S'| - {{\,\mathrm{\textsf {sl}\mathrm }\,}}{S'}$ (Regularity Preservation Lemma 10.10).

Since $\textsf {S} _{\textsf {triv} }$ is regular and $\dim \Gamma (\textsf {S} _{\textsf {triv} }) = 3 = |{\textsf {V} }\textsf {S} _{\textsf {triv} }| - {{\,\mathrm{\textsf {sl}\mathrm }\,}}{\textsf {S} _{\textsf {triv} }}$, this immediately yields an inductive argument for Theorem 10.1. For the proof of (C), we consider the perfect coarsener *K* of $S'$ whose isolation leads to *S*, and a height function $\omega _1$ that realizes *S*. First, we show that $\dim \Gamma (S')>\dim \Gamma (S)$, and, therefore, a height function $\omega '\in \Gamma (S') \setminus \Gamma (S)$ exists. According to (B), the $\omega '$-labeling assigns the same label to all edges incident to *K*, and since $\omega ' \notin \Gamma (S)$, this label cannot be 0. Hence, either $\omega '$ or $-\omega '$ assigns constant $\ominus$, and it can be used for a controlled perturbation $\omega _1 + \varepsilon \omega '$ which realizes $S'$. We will now carefully work out these steps.

### Valid $\{\oplus ,\ominus ,0\}$-Edge Labelings

#### Definition 10.6

**Fig. 33:**
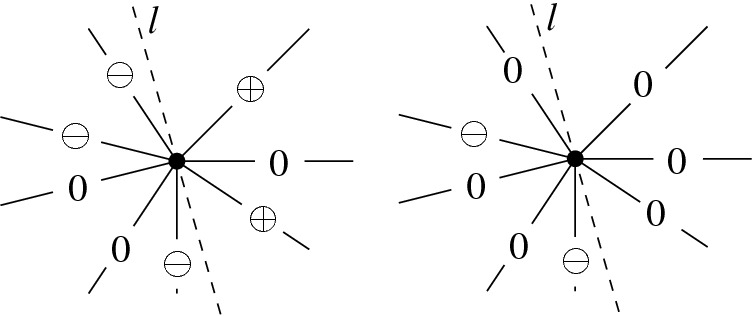
Patterns prohibited in valid labelings

Let *S* be a subdivision. Given a labeling $\alpha :{\textsf {E} ^\circ }S \rightarrow \{\oplus ,\ominus ,0\}$, we call an inner point $p\in {\textsf {V} ^\circ }S$ $\alpha$-*pointed*, if $\alpha$ is not constant 0 on the edges incident to *p*, and if there is a line through *p* that has all $\oplus$-labeled edges incident to *p* strictly on one side and all $\ominus$-labeled edges incident to *p* strictly on the other side of this line. (We do not require that both $\oplus$- and $\ominus$-labeled edges incident to *p* exist.) We call $\alpha$ a *valid labeling of* ${\textsf {E} ^\circ }S$ if no point in ${\textsf {V} ^\circ }S$ is $\alpha$-pointed (Fig. 33).

For example, for an inner point of degree $3$ in a subdivision, a valid labeling must assign the same label to its three incident edges. We can now prove (A) above.

#### Lemma 10.7

Let $\omega$ be a height function compliant with subdivision *S*. Then the $\omega$-labeling of *S* is a valid labeling of ${\textsf {E} ^\circ }S$.

#### Proof

Let $p \in {\textsf {V} ^\circ }S$ and suppose there is a line $\ell$ through *p* that has all $\oplus$-labeled edges incident to *p* on one side, and all $\ominus$-labeled edges incident to *p* on the other side. Sweep a vertical plane *h* parallel to $\ell$ in $\mathbb {R}^3$ over *p* and observe its intersection with the $\omega$-lifting *f*. On the side of the $\oplus$-labeled edges, this intersection must be a locally concave function, on the side of the $\ominus$-labeled edges a locally convex function. Consequently, it has to be locally linear at the point when *h* contains *p* and $\ell$. Now it follows that *f* must be locally linear around *p* and all edges incident to *p* must be flat. $\square$

#### Lemma 10.8

Let *K* be a perfect coarsener in a subdivision *S*. In every valid $\{\oplus ,\ominus ,0\}$-labeling of ${\textsf {E} ^\circ }S$, the edges $E_K$ incident to *K* all get the same label.

#### Proof

We plan to prove the following.

**Claim**. *With reference to the orientation process in the proof of Lemma* 8.6, *after the second phase, in any valid labeling, the edges incident to a candidate component of* $G^*$ *get the same label.*

It is not obvious from the proof of Lemma 8.6 that every perfect coarsener is identified by the three phase process. In order to close this gap (from the claim to the assertion of the lemma), isolate *K* in *S* obtaining a subdivision $S'$ with a region *r* containing the points in *K*. Let $V_r$ be the vertices of this region, i.e., . Now consider the subgraph $S_r$ of *S* induced by $V_r\cup K$. *K* is a perfect coarsener of $S_r$ whose isolation yields the trivial subdivision of $V_r \cup K$. It is the only coarsener of $S_r$ and ${{\,\mathrm{\textsf {sl}\mathrm }\,}}{S_r} = |{\textsf {V} }S_r|-4$. Therefore, the procedure in the proof of Lemma 8.6 must identify *K* as a candidate component after the second phase. We establish the claim by showing the following invariant in the process during the second phase:

- For every candidate component *K*, the edges $E_K$ incident to *K* obtain the same label in any valid labeling.
- An edge gaining a new orientation in the second phase and its witnesses obtain the same label in any valid labeling.

At the end of the first phase, a point with indegree $3$ in $\vec {S}$ has actually degree $3$ in *S*, and therefore any valid labeling must give the same label to all incident edges. Since in a candidate component *K*, all points have indegree $3$ in $\vec {S}$ (i.e., at this point, have degree $3$ in *S*) and since a connected component is connected [sic!], it easily follows that all edges incident to a candidate component must have the same label in any valid labeling.

During the second phase, a newly oriented edge and its witnesses are part of the edges incident to a candidate component. Hence, given (a), invariant (b) is maintained after an orientation step of phase 2. We are left to show that (a) is preserved. Consider a point *p* of a candidate component. It must have indegree $3$, all incident edges are either ingoing or witnesses for an ingoing edge (otherwise, indegree $3$ is impossible); we know that each bundle of an ingoing edge and its witnesses have the same label, and such a bundle can be separated from the other incident edges by a line through the given point *p* (this is why the edge was oriented in phase 2). Hence, due to a simple consideration, any valid labeling must assign the same label to all incident edges. (The simple consideration: Suppose a single bundle is labeled $\oplus$, then this bundle can be separated from the other two bundles by a line, contradiction. Suppose exactly two bundles are labeled $\oplus$, then the remaining bundle can be separated from these two $\oplus$-labeled bundles by a line, which is a contradiction. Hence, if any incident edge is labeled $\oplus$, then all incident edges must be labeled $\oplus$. Similarly, for $\ominus$.) This completes the proof of the claim, and thus of the lemma. $\square$

Now, with Lemma 10.7 we immediately get property (B).

#### Lemma 10.9

If $\omega$ is an *S*-compliant height function, then the $\omega$-labeling is constant on any set of edges incident to a perfect coarsener of *S*.

### The Regularity Preservation Lemma

#### Lemma 10.10

(Regularity Preservation). Let $S_1$ be a regular subdivision with $\dim \Gamma (S_1) = |{\textsf {V} }S_1| - {{\,\mathrm{\textsf {sl}\mathrm }\,}}{S_1}$. If $S_0 \prec _1S_1$, then $S_0$ is regular and $\dim \Gamma (S_0) = |{\textsf {V} }S_0| - {{\,\mathrm{\textsf {sl}\mathrm }\,}}{S_0}$.

#### Proof

Let $\omega _1 \in \mathbb {R}^{{\textsf {V} }S_1}$ be a height function that realizes $S_1$.

*Case 1*. $S_1$ is obtained from $S_0$ by adding a single point $p \in P^\circ \setminus {\textsf {V} ^\circ }S_{0}$. Then  realizes $S_0$ and $S_0$ is regular. We have . For $\omega \in \Gamma (S_1)$, the value of $\omega _p$ (*p* the added point) is determined by , i.e., $\dim \Gamma (S_1) = \dim \Gamma (S_0)$. Therefore,

$$\begin{aligned} \dim \Gamma (S_0) = |{\textsf {V} }S_1| - {{\,\mathrm{\textsf {sl}\mathrm }\,}}{S_1} = (|{\textsf {V} }S_0| + 1)-({{\,\mathrm{\textsf {sl}\mathrm }\,}}{S_0} + 1) = |{\textsf {V} }S_0| - {{\,\mathrm{\textsf {sl}\mathrm }\,}}{S_0}. \end{aligned}$$

*Case 2*. $S_1$ is obtained from $S_0$ by removing a single unlocked edge or by isolating a perfect coarsener in $S_0$. We have ${\textsf {V} }S_1={\textsf {V} }S_0$. The set $E^* := \textsf {E} S_0 \setminus \textsf {E} S_1$ is either a single unlocked edge or the set $E_K$ of edges incident to a perfect coarsener *K* in $S_0$. Let $r^*$ be the region in $S_1$ generated by the removal of the edges in $E^*$.

We have $\Gamma (S_0) \supseteq \Gamma (S_1)$ and (with Lemma 10.3(iii) and ${{\,\mathrm{\textsf {sl}\mathrm }\,}}{S_0} = {{\,\mathrm{\textsf {sl}\mathrm }\,}}{S_1} - 1$)

$$\begin{aligned} \dim \Gamma (S_0) \ge |{\textsf {V} }S_0| - {{\,\mathrm{\textsf {sl}\mathrm }\,}}{S_0} = |{\textsf {V} }S_1| - {{\,\mathrm{\textsf {sl}\mathrm }\,}}{S_1} + 1 = \dim \Gamma (S_1) + 1. \end{aligned}$$

Therefore, there must exist $\omega ' \in \Gamma (S_0) \setminus \Gamma (S_1)$, a height function that is not flat on $r^*$ and there is an edge in $E^*$ that is not flat in the $\omega '$-lifting of $S_0$. In that case, all edges in $E^*$ are mountains or all are valleys (trivially true if $|E^*|=1$, otherwise by Lemma 10.9). Let us suppose that the $\omega '$-labeling is constant $\ominus$ on $E^*$ (if not switch to $-\omega '$). Now, for any sufficiently small positive $\varepsilon \in \mathbb {R}$, the height function $\omega _0:= \omega _1 + \varepsilon \omega '$ is compliant with $S_0$ and all inner edges in $S_0$ are valleys in the $\omega _0$-lifting of $S_0$ ($\varepsilon$ has to be small enough such that all valleys in the $\omega _1$-lifting remain valleys in $\omega _0$; this is a familiar operation, see [24, Lemma 2.3.16]). This establishes that $S_0$ is regular.

We are left to show that $\dim \Gamma (S_0) = |{\textsf {V} }S_0| - {{\,\mathrm{\textsf {sl}\mathrm }\,}}{S_0}$, or, equivalently, $\dim \Gamma (S_0)=\dim \Gamma (S_1) +1$. This holds, if for any two $\omega '$, $\omega ''$ in $\Gamma (S_0) \setminus \Gamma (S_1)$ there exists $a \in \mathbb {R}$ and $\omega \in \Gamma (S_1)$ such that $\omega '=a\omega '' +\omega$. Suppose that all edges in $E^*$ are valleys in $\omega '$, and all edges in $E^*$ are mountains in $\omega ''$ (switch signs, if necessary). Now consider $\omega _t := (1-t)\omega ' + t \omega ''$, $t \in [0,1]$. There must be a value *t* in (0, 1) where some edge in $E^*$ is flat in the $\omega _t$-lifting, but then all edges have to be flat and $\omega _t \in \Gamma (S_1)$ (Lemma 10.9). We have shown

$$\begin{aligned} \omega ' = -\frac{t}{1-t}\omega '' + \frac{1}{1-t}\omega _t,\qquad \omega _t \in \Gamma (S_1). \end{aligned}$$

$$\square $$

### Proof of Theorem 10.1

#### Proof

If $S \prec ^*_1\textsf {S} _{\textsf {triv} }$ then there is a sequence

$$\begin{aligned} S=S_0 \prec _1S_1 \prec _1\ldots \prec _1S_\ell = \textsf {S} _{\textsf {triv} }. \end{aligned}$$

$\textsf {S} _{\textsf {triv} }$ is regular, ${{\,\mathrm{\textsf {sl}\mathrm }\,}}{\textsf {S} _{\textsf {triv} }} = n-3$ and $\Gamma (\textsf {S} _{\textsf {triv} }) = \Lambda (\textsf {S} _{\textsf {triv} })$, of dimension $3 = n - {{\,\mathrm{\textsf {sl}\mathrm }\,}}{\textsf {S} _{\textsf {triv} }} = |\textsf {V} {\textsf {S} _{\textsf {triv} }}|-{{\,\mathrm{\textsf {sl}\mathrm }\,}}{\textsf {S} _{\textsf {triv} }}$. Along with Lemma 10.10 we have an inductive argument that $S_0 = S$ is regular. $\square$

We immediately get that successive perfect refinements of a subdivision $S^*$ fill the regions of $S^*$ with locally regular subdivisions.

#### Definition 10.11

(*restriction of subdivision*). Let $S\preceq S'$ be subdivisions, and let $r\in {\textsf {R} }S'$. Then the *restriction of* *S* *to* *r*, , is the subgraph of *S* induced by ${\textsf {V} }S\cap \overline{r}$ ($\overline{r}$ the closure of *r*).

#### Corollary 10.12

Let $S \prec ^*_1S'$. For $r \in {\textsf {R} }S'$, we have  and  is a regular subdivision of ${\textsf {V} }r$.

Let us conclude this section with the remark, that successive perfect coarsening starting from a subdivision is a non-deterministic process, that may—even for the same subdivision—lead to $\textsf {S} _{\textsf {triv} }$ or not (see Fig. 21 at the beginning of Sect. 8).

## Implications of Regularity Preservation

### Covering the Bistellar Flip Graph with Polytopes

#### Theorem 11.1

The edge set of the bistellar flip graph of *P* can be covered by subgraphs isomorphic to 1-skeletons of $(n-3)$-polytopes (which are products of secondary polytopes).

#### Proof

Given an edge $\{T,T[x]\}$ of the bistellar flip graph, let *S* be a subdivision with $T_{\pm x} \prec ^*_1S$ and ${{\,\mathrm{\textsf {sl}\mathrm }\,}}{S} = n-3$ (Corollary 8.8). For every region *r* of *S*, the subdivisions , , and  are regular subdivisions of ${\textsf {V} }r$; this holds, since $T \prec _1T_{\pm x}$ and $T[x] \prec _1T_{\pm x}$ (Lemma 7.8), thus $T\prec ^*_1S$ and $T[x]\prec ^*_1S$.

Now consider the product of polytopes (see [39])

$$\begin{aligned} \prod _{r \in {\textsf {R} }S} \Sigma \text{- }\textsf {poly} ({\textsf {V} }r), \end{aligned}$$

where $\Sigma \text{- }\textsf {poly} (A)$ denotes the secondary polytope of $A \subseteq P$ [24], see also Sect. 1.4, Theorem 1.11. The dimension of this product is $\sum _r (|{\textsf {V} }r|-3) = {{\,\mathrm{\textsf {sl}\mathrm }\,}}{S} = n-3$. Its faces correspond to the refinements $S'$ of *S* such that for each region *r* of *S*,  is regular, i.e., this includes *T* and *T*[*x*] (as vertices), and $T_{\pm x}$ (as edge) (Corollary 10.12). This completes the argument. $\square$

**Fig. 34:**
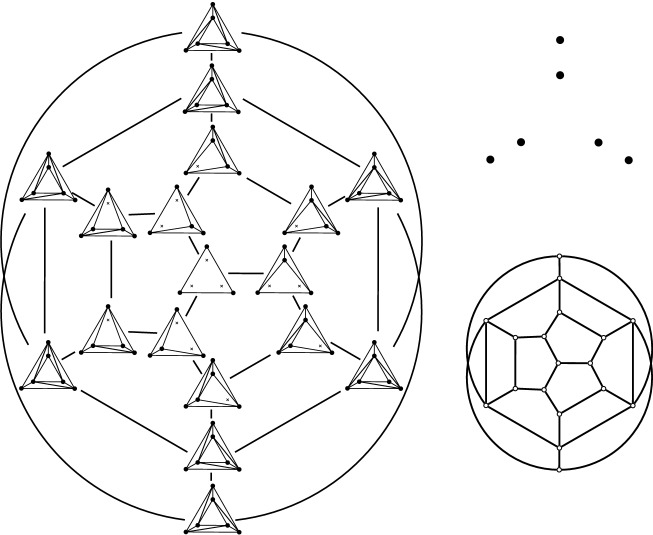
The bistellar flip graph of the mother-of-examples configuration

**Fig. 35:**
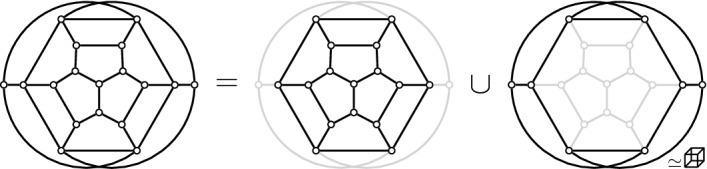
The bistellar flip graph of the mother-of-examples configuration as the union of the graphs of two 3-polytopes

### Sets with All Triangulations Regular

We give characterizations of point sets for which all triangulations are regular (as, e.g., it is the case for point sets in convex position). In particular, we show that this can be easily read off the height of the partial order $\preceq$. In a first step we prove that property to be equivalent to requiring that all subdivisions are regular.

#### Lemma 11.2

All subdivisions are regular iff all triangulations are regular.

#### Proof

The direction ($\Rightarrow$) is obvious. For ($\Leftarrow$) it suffices to show that every non-regular subdivision *S* with ${{\,\mathrm{\textsf {sl}\mathrm }\,}}{S} > 0$ has a direct refinement which is not regular.

Case 1. *S* has a bystander $p \in {\textsf {V} }^{\textsf {by}}{S}$. Clearly, the direct refinement $({\textsf {V} }S \setminus \{p\}, \textsf {E} S)$ is not regular iff *S* is not regular.

Case 2. *S* has no bystander. Since $\textsf {sl} ~ {S}>0$, *S* must have an active region $r^*$ which is a *k*-gon for $k\ge 4$. Choose two crossing diagonals $e_0$ and $e_1$ in $r^*$ and consider the subdivisions $S_i := ({\textsf {V} }S, \textsf {E} S \cup \{e_i\})$, $i=0,1$. We want to show that if *S* is not regular, then at least one of $S_0$ and $S_1$ is not regular. So let us suppose that, for $i=0,1$, $\omega _i \in \mathbb {R}^{{\textsf {V} }S}$ is a height function realizing $S_i$ (as a regular subdivision) and, for $t \in [0,1]$, consider the convex combination $\omega _t := (1-t)\omega _0 + t\omega _1$.

We say that a height function $\omega$ *respects* region *r* in subdivision *S*, if $\omega$ is linear on ${\textsf {V} }r$ and all points in ${\textsf {V} }S^{(\omega )} \setminus {\textsf {V} }r^{(\omega )}$ lie strictly above the plane spanned by ${\textsf {V} }r^{(\omega )}$. Clearly, $\omega$ realizes *S* iff it respects all regions $r \in {\textsf {R} }S$. Moreover, if two height functions respect a region, then all convex combinations do.

It follows that $\omega _t$ respects all regions in ${\textsf {R} }S$ except for $r^*$, since these are regions both in $S_0$ and $S_1$. We have that $e_0^{(\omega _0)}$ lies below $e_1^{(\omega _0)}$ (as segments in the lifting in $\mathbb {R}^3$), while $e_1^{(\omega _1)}$ lies below $e_0^{(\omega _1)}$ and, therefore, there must be a $t \in (0,1)$, where $e_0^{(\omega _t)}$ and $e_1^{(\omega _t)}$ intersect (in the lifting in $\mathbb {R}^3$). For that value of *t*, $\omega _t$ is linear on ${\textsf {V} }r^*$. Moreover, all edges in  are valleys in the $\omega _t$-lifting, since these are valleys both in the $\omega _0$-lifting and the $\omega _1$-lifting (and that property is preserved for all convex combinations of $\omega _0$ and $\omega _1$). Hence, $\omega _t$ realizes *S* and we have a contradiction. $\square$

We recall the definition of the height of an element in a partial order, and of the height of the partial order.

#### Definition 11.3

(*height*). The *height*, $\textsf {h} S$, *of* a subdivision  *S* (in the partial order $\preceq$) is recursively defined: (a) If *S* is a triangulation, then $\textsf {h} S : = 0$, and (b) if *S* is not a triangulation, then $\textsf {h} S := 1+ \max _{S' \prec _{\textrm{dir}}S} \textsf {h} S'$. (Equivalently, $\textsf {h} S$ is the size of the longest $\preceq$-chain ending in *S* minus $1$.) We let $\textsf {h} _\textsf {max} = \textsf {h} _\textsf {max} (P)$ be the maximum height of any subdivision of *P* (i.e., $\textsf {h} _\textsf {max} = \textsf {h} \textsf {S} _{\textsf {triv} }$).

#### Theorem 11.4

The following five conditions are equivalent.

(i) All triangulations are regular.
(ii) All subdivisions are regular.
(iii) $$\textsf {h} _\textsf {max} = n-3$$.
(iv) $$\prec _{\textrm{dir}}=~\prec _1$$.
(v) $$\textsf {h} = \textsf {sl} $$.

#### Proof

For (i) $\Leftrightarrow$ (ii) see Lemma 11.2. For the rest we show the implication cycles

$$\begin{aligned}{} & {} \text {all subdivisions are~ regular} {\mathop {\Rightarrow }\limits ^{(a) }}\\ \textsf {h} _\textsf {max}{} & {} {=}~ n-3~{\mathop {\Rightarrow }\limits ^{(b) }}~\prec _{\textrm{dir}}~ = \prec _1~{\mathop {\Rightarrow }\limits ^{(c) }}\text {all subdivisions are regular},\\ {}{} & {} {{\textsf {h} _\textsf {max} = n-3 {\mathop {\Rightarrow }\limits ^{\mathrm {(b)}}} \prec _{\textrm{dir}}= \prec _1{\mathop {\Rightarrow }\limits ^{\mathrm {(d)}}} \textsf {h} = \textsf {sl} {\mathop {\Rightarrow }\limits ^{\mathrm {(e)}}} \textsf {h} _\textsf {max} = n-3}.} \end{aligned}$$

- *All subdivisions are regular* $\Rightarrow$ $\textsf {h} _\textsf {max} = n-3$.   This is well known and discussed, e.g., in “Twelve proofs of non-regularity” in [24, Sect. 7.1.2, (6)]: On the one hand, if all subdivisions are regular, they all correspond to faces of the secondary polytope, an $(n-3)$-polytope where every chain of proper faces (excluding the empty face and the polytope itself) has size at most $n-3$. On the other hand, if $\textsf {h} _\textsf {max} >n-3$, that gives a chain of size exceeding $n-3$ of non-trivial subdivisions. (Note here: There is a maximum chain of size $1+\textsf {h} _\textsf {max}$ in the $\preceq$-partial order. If we remove the trivial subdivision (not corresponding to a proper face), we get still a chain of length $\textsf {h} _\textsf {max}$.)
- $\textsf {h} _\textsf {max} =n-3$ $\Rightarrow$ $\prec _{\textrm{dir}}~=\prec _1$.   Consider a maximal chain $S_0 \prec _{\textrm{dir}}S_1 \prec _{\textrm{dir}}\ldots \prec _{\textrm{dir}}S_m$; because of maximality, $S_0$ is a triangulation (of slack 0), and $S_m = \textsf {S} _{\textsf {triv} }$ (of slack $n-3$). We know that ${{\,\mathrm{\textsf {sl}\mathrm }\,}}{S_i} \le {{\,\mathrm{\textsf {sl}\mathrm }\,}}{S_{i-1}} +1$ (Lemma 11.5 below) with equality iff $S_{i-1} \prec _1S_i$. It follows that $m \ge n-3$. Moreover, if $m=n-3$ (which is given if $\textsf {h} _\textsf {max} = n-3$), then $S_{i-1}\prec _1S_i$ for all $i=1,2,\ldots ,n-3$. Since every pair $S' \prec _{\textrm{dir}}S$ is part of a maximal chain, the claim follows.
- $\prec _{\textrm{dir}}~=\prec _1$ $\Rightarrow$ *all subdivisions are regular.*   Every subdivision *S* has a chain of direct coarsenings to $\textsf {S} _{\textsf {triv} }$. If every direct coarsening is a perfect coarsening, this shows $S \prec ^*_1\textsf {S} _{\textsf {triv} }$ and therefore *S* is regular (Theorem 10.1).
- $\prec _{\textrm{dir}}~ = \prec _1$ $\Rightarrow$ $\textsf {h} = \textsf {sl}$.   For proving $\textsf {h} S = {{\,\mathrm{\textsf {sl}\mathrm }\,}}{S}$, we can proceed by induction on the height of *S*, where the induction basis holds without assumptions. With the assumption of $\prec _{\textrm{dir}}= \prec _1$ and with the induction hypothesis
  $$\begin{aligned}{} & {} \textsf {h} S = 1+ \max _{S' \prec _{\textrm{dir}}S} \textsf {h} S'= 1+ \max _{S' \prec _1S} \textsf {h} S'= 1+ \max _{S' \prec _1S} {{\,\mathrm{\textsf {sl}\mathrm }\,}}{S'}\\{} & {} \quad \quad = 1+\max _{S' \prec _1S} ({{\,\mathrm{\textsf {sl}\mathrm }\,}}{S}-1)= {{\,\mathrm{\textsf {sl}\mathrm }\,}}{S} \end{aligned}$$
  For the last equality, note that for a subdivision that is not a triangulation, a perfect refinement can always be obtained by simply adding an edge, or by involving a bystander as a point of degree $3$. If there is no edge to add and if there is no bystander, we have a triangulation.
- $$\textsf {h} = \textsf {sl} {}$$ $$\Rightarrow $$ $$\textsf {h} _\textsf {max} = n-3$$.   If $$\textsf {h} = \textsf {sl} {}$$, then $$\textsf {h} _\textsf {max} = \textsf {h} \textsf {S} _{\textsf {triv} }={{\,\mathrm{\textsf {sl}\mathrm }\,}}{\textsf {S} _{\textsf {triv} }} = n-3$$. $$\square $$

### More Properties of Coarseners

We derive two more properties of prime and perfect coarseners, Lemma 11.5, which we used in the proof of Theorem 11.4 above, and Lemma 11.6, which we will need in Sect. 11.4 below.

#### Lemma 11.5

${{\,\mathrm{\textsf {inc}\mathrm }\,}}{U} \le 1$ for every prime coarsener *U* in a subdivision  *S*.

#### Proof

Let *r* be the region in $S'$ obtained by removing from *S* the edges $E_U$ incident to *U*. The subgraph of *S* induced by *U* is connected (Observation 8.4 (iv)), that is, all points of *U* have to lie in the same region of $S'$. We consider the restriction  (Definition 10.11), a subdivision of . Isolating *U* in  yields , the trivial subdivision of . Therefore,that is, . On the one hand, *U* is the only coarsener of  (since *U* is prime and it exhausts all inner points in ). On the other hand, the Coarsening Lemma 8.6 guarantees , i.e., ${{\,\mathrm{\textsf {inc}\mathrm }\,}}{U}$ (perfect) coarsenings of . Therefore, ${{\,\mathrm{\textsf {inc}\mathrm }\,}}{U} \le 1$. $\square$

#### Lemma 11.6

A prime coarsener *U* inducing a tree in its subdivision *S* is perfect.

#### Proof

Let $k:= |U|$ and let $\ell$ be the number of edges in ${\textsf {E} ^\circ }S$ that are incident to exactly one point in *U*. We have $|E_U| = (k-1)+\ell$. Since every point in *U* has degree at least $3$, we have $|E_U| \ge ({3k + \ell })/{2}$. Hence, $(k-1)+\ell \ge ({3k + \ell })/{2}$, i.e., $\ell \ge k+2$. Now ${{\,\mathrm{\textsf {inc}\mathrm }\,}}{U} = |E_U| - 2|U| = (k-1)+\ell - 2k = \ell - k - 1 \ge 1$, i.e., by Lemma 11.5, ${{\,\mathrm{\textsf {inc}\mathrm }\,}}{U} = 1$.^2^$\square$

### Large Minimal Sets with Non-Regular Triangulations—Proof of Theorem 1.14

#### Observation 11.7

If $P'\subseteq P$ and $P'$ has non-regular triangulations, then *P* has non-regular triangulations.

Given a set *P* with non-regular triangulations, is there always a small subset $P'$ of *P* that witnesses this fact? This is a question asked by Francisco Santos^3^ (see also Sect. 12.4 for a computational complexity related motivation for this question). An equivalent reformulation is: How large can minimal sets *P* with non-regular triangulations be? (Here, “minimal” means that every proper subset of *P* has only regular triangulations.) Santos describes such a minimal set of eight points and conjectures this to be the largest example of such a minimal set. We will show that there exist such minimal sets of arbitrarily large (even) size.

**Fig. 36:**
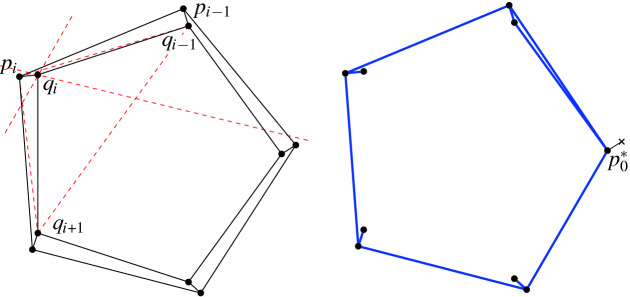
A twisted double-gon of ten points with subdivision  $S^\Box$ (left). Subset $P^*$ (case $p_0^* = q_0$) of a twisted double-gon (right)

#### Definition 11.8

(*twisted double-gon*). A set *P* of *n* points in general position, $n=2k$ even, is called a *twisted double-gon* if the following holds.

(I) $$|{{{\,\mathrm{\textsf {xtr}\mathrm }\,}}P}| = k$$.

Let $p_0,p_1,\ldots ,p_{k-1}$ be a counter-clockwise numbering of ${{\,\mathrm{\textsf {xtr}\mathrm }\,}}{P}$ along the boundary of the convex hull of *P*.

(II) The set $Q:=P^\circ$ of inner points is in convex position.

There is a numbering $q_0,q_1,\ldots ,q_{k-1}$ of *Q* following the order along the boundary of the convex hull of *Q* such that for all *i*, $0 \le i \le k-1$, it holds:

(III) $q_i$ is extreme in $P \setminus \{p_i\}$.
(IV) $q_i$ is extreme in $P \setminus \{p_{i-1},q_{i-1}\}$.
(V) $q_i$ lies in the triangle $q_{i-1}p_iq_{i+1}$.

Figure 36 indicates that such twisted double-gons exist for all even $n\ge 6$ (these are not double-circles, see e.g. [2]; actually, double-circles have only regular triangulations). For $n=6$, this is the mother-of-examples configuration (Sect. 10.2). Here are a few simple observations.

#### Observation 11.9

Let *P* be a twisted double-gon, with notation as in Definition 11.8.

(i) The graph $S^\Box :=(P,{\textsf {E} _\textsf {hull} }\cup \{\{q_i,p_i\},\{q_i,q_{i+1~mod ~k}\}\mid i=0,1,\ldots ,k-1\})$ is a subdivision (uses (II) and (V)). Its slack is $n-3$.
(ii) If $q_i$ is involved in a subdivision of a subset of *P* where it is not extreme, it is connected to $p_i$ (by (III)) and to at least one of $\{p_{i-1},q_{i-1}\}$ (by (IV)).

Here comes the lemma that shows that twisted double-gons constitute examples showing Theorem 1.14.

#### Lemma 11.10

If *P* is a twisted double-gon, then

(i) *P* has a non-regular triangulation, and
(ii) any proper subset of *P* has only regular triangulations.

#### Proof

(i) Since ${{\,\mathrm{\textsf {sl}\mathrm }\,}}{S^\Box } = n-3$, we have $\textsf {h} S \ge n-3$ (by Lemma 11.5), therefore $\textsf {h} \textsf {S} _{\textsf {triv} } > n-3 = {{\,\mathrm{\textsf {sl}\mathrm }\,}}{\textsf {S} _{\textsf {triv} }}$ and $\textsf {h} \ne \textsf {sl}$. Theorem 11.4 implies that *P* has non-regular triangulations. Note that we do not claim that $S^\Box$ is a non-regular subdivision; this depends on the concrete coordinates of the point set *P*.
(ii) We remove $p_0$ or $q_0$ from *P*, we denote the resulting set by $P^*$ with $p_0^*$ the point among $p_0$ and $q_0$ remaining in $P^*$, see Fig. 36, right. If we can show that all triangulations of $P^*$ are regular, then the proof is complete (by symmetry and Observation 11.7).

It is enough to show that any prime coarsener *U* of any subdivision *S* of $P^*$ is perfect (by Lemma 11.5 this is equivalent to ${{\,\mathrm{\textsf {inc}\mathrm }\,}}{U}=|E_U|-2|U|\ge 1$), since then $\prec _{\textrm{dir}}~=\prec _1$ and Theorem 11.4 can step in. So let us consider such a prime coarsener *U*, let $E^in _U$ be the edges in *S* connecting two points in *U*, and let $E^out _U$ be the set of edges in *S* connecting a point in *U* to a point in ${\textsf {V} }S\setminus U$. We have $E_U=E^in _U\cup E^out _U$.

- The subgraph of *S* induced by *U* has to be connected (since *U* is prime, Observation 8.4 (iv)), hence $|E^in _U| \ge |U|-1$.
- If the subgraph of *S* induced by *U* is a tree, then, by Lemma 11.6, *U* is a perfect coarsener.

So let us assume that *U* does not span a tree, which implies $|E^in _U|\ge |U|$.

(c) Every point in *U* has to connect to at least one point in ${\textsf {V} }S\setminus U$. This holds, since $U\subseteq Q\setminus \{q_0\}$ and $q_i\in U$, $i \ge 1$, has to connect to $p_i$ (Observation 11.9 (ii)). Hence, $|E^out _U| \ge |U|$.

At this point we have already shown that $|E_U| = |E^in _U| + |E^out _U| \ge 2|U|$. Therefore, ${{\,\mathrm{\textsf {inc}\mathrm }\,}}{U}\ge 0$, and *U* is perfect unless $|E^in _U| = |U|$ and $|E^out _U| = |U|$.

(d) Let $j := \min {\{i\in \mathbb {N}\mid i\ge 1,~q_i\in U\}}$. If $j=1$, i.e., $q_1\in U$, then $q_1$ connects to $p_1$ and $p_0^*$. Hence, $|E^out _U|\ge |U|+1$. If $j \ge 2$, then $q_j$ has to connect to $p_j$ and $p_{j-1}$ (by Observation 11.9 (ii)), since $q_{j-1}$ is not available). Hence, $|E^out _U| \ge |U|+1$.

We can conclude that *U* has to be perfect. $\square$

## Discussion and Open Problems

We conclude by briefly discussing some questions that naturally arise in connection with this work.

### The Min-Degree Bound for Small Sets

We have put, with no success, some effort into getting rid of the “*P* large enough” condition in the min-degree bound of Theorem 1.7 (i) for the edge flip graph. In fact, the min-degree bound was our initial result, and the other results have emerged in the quest to get rid of the “*P* large enough” condition. As indicated right after Theorem 1.7 in Sect. 1.1, the min-degree bound holds for all point sets with up to ten points [12]. Since very small point sets initially seemed the most likely candidates for counterexamples (for instance, because Lemma 3.1 fails for a set of six points, Fig. 10), we conjecture that the “*P* large enough” condition can indeed be removed.

### General vs. Special Position

**Fig. 37:**
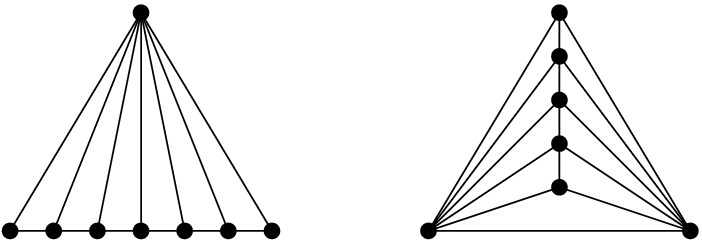
Point sets with a unique full triangulation

When abandoning the general position assumption, i.e., allowing collinearities of three or more points, the situation changes significantly (actually, even repeated points have to be considered [24]). The edge flip graph may degenerate to a single vertex even for large point sets (Fig. 37).

**Fig. 38:**
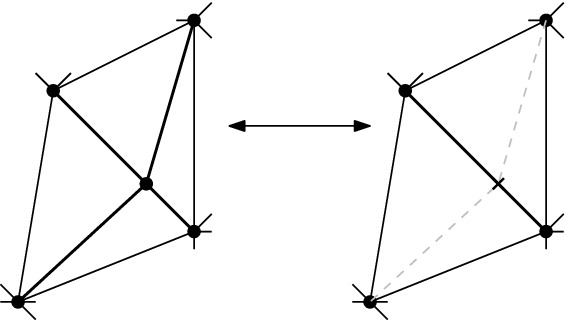
A flip in the presence of collinearities

For bistellar flips, new flips as shown in Fig. 38 come into play. Theorem 1.8 ([25], see also [24, Thm. 3.4.9]) still holds in this situation and $n-3$ bistellar flips are guaranteed. We are currently investigating with Grelier to what extent our methods generalize from general to special position towards $(n-3)$-vertex connectivity of the bistellar flip graph, which is by no means obvious.

### Stably Regular Triangulations

It might be interesting to understand, which regular triangulations are captured by Theorem 10.1, i.e., partial triangulations in the $\prec ^*_1$-lower closure of $\textsf {S} _{\textsf {triv} }$. From the discussion in Sect. 10.2 (and Fig. 32) we recall one reason why this condition for regularity cannot possibly be necessary: It depends only on the order type (or oriented matroid) of *P*, while regularity depends on the coordinates of the point set. So let us define a partial triangulation *T* of *P* *stably regular*, if it is regular on any realization of the order type of *P*. (A careful formulation of “regular on any realization of the order type” is: Whenever there is a bijective mapping $p\mapsto p'$ from *P* to a set $P'$ which preserves orientation of triples, then the partial triangulation $T'$ of $P'$ obtained by ${\textsf {V} }T' = \{p'~|p \in {\textsf {V} }T\}$ and $\textsf {E} T' = \{\{p',q'\} \mid \{p,q\} \in \textsf {E} T\}$ is also regular.) Note that if we can move the points in *P* freely while preserving *T* as a triangulation, then we can turn any triangulation into a regular one. This follows basically from Steinitz’s Theorem (see [39, Theorem 4.1]).

We believe that Theorem 10.1 goes as far as one can go with an order type based condition.

#### Conjecture 12.1

A triangulation $T \in {{\mathcal {T}}}_{\textsf {part} }(P)$ is stably regular iff $T \prec ^*_1\textsf {S} _{\textsf {triv} }$.

One might ask whether the subdivisions in the $\prec ^*_1$-down set of $\textsf {S} _{\textsf {triv} }$ correspond to the face lattice of a polytope. In general, this is not the case, it is not even an abstract polytope, (see [27, Chapter 2A] for abstract polytopes): We can choose ${{\,\mathrm{\textsf {sl}\mathrm }\,}}{S}$ as a rank function, have $\textsf {S} _{\textsf {triv} }$ as maximal/greatest element, and add an empty face as minimal/least element of rank $-1$. All flags (i.e., maximal chains) have the same size. But we fail on the so-called *diamond axiom*: “If *S* is a subface of $S'$ with the ranks differing by $2$, then there are exactly two faces strictly between *S* and $S'$.” Figure 21 shows an example where this axiom fails: *S* and $\textsf {S} _{\textsf {triv} }$ in this figure differ by $2$ in slack, but there is only one subdivision strictly in between the two (in the $\prec _1$-order).

### Minimal Sets with Non-Regular Triangulations

In Sect. 11.4 we have identified arbitrarily large minimal sets with non-regular triangulations. Given that such sets of arbitrary size exist, it is not clear how to check efficiently whether a set allows only regular triangulations. A first step would be to understand all minimal sets with non-regular triangulations. It is possible that twisted double-gons as defined in Definition 11.8 are the only such sets.

Let us mention here that the problem of deciding whether a given triangulation is regular can be answered in polynomial time via linear programming.

### The Fundamental Group

Consider the set of closed walks in an edge or bistellar flip graph, starting in a fixed triangulation $T^*$. Consider a cycle $c=(T_0,T_1,\ldots ,T_{k-1})$, $k \ge 2$, in the flip graph, i.e., $T_i$ and $T_{i + 1~mod ~k}$ are adjacent triangulations for $0\le i<k$. An *insertion of c* adds *c* before an occurrence of $T_0$ in a closed walk, and a *deletion of c* removes *c* if it occurs before $T_0$ in a closed walk. Let us consider two closed walks equivalent, if they can be obtained from each other by insertion and deletion of cycles of length 2, or of cycles spanned by the refinements of a subdivision of slack $2$ (these are 4- or 5-cycles, one can show that these are exactly the 4- and 5-cycles, both for the edge and the bistellar flip graph.). Lubiw et al. [26] show that in the *edge* flip graph all closed walks containing a triangulation $T^*$ are equivalent to the trivial walk $(T^*)$; in other words, the fundamental group of the complex spanned by slack 2 subdivisions (which correspond to the 2-faces of the flip complex) is trivial. This is the decisive step in proving the orbit conjecture of [7] (see discussion in Sect. 1.5). It is natural to ask, whether the analogous result holds for the bistellar flip graph, or, more generally, whether there is a bistellar analogue of the flip complex.

### Higher Dimensions

Flip graphs can be defined on triangulations in higher dimensions, i.e., tetrahedralizations in $\mathbb {R}^3$, etc. , see [24], but even for sets of points in convex and general position in 3-space it is open, whether these graphs are always connected. As mentioned before, because of the Local Menger Lemma 2.3, we were able to show high vertex connectivity without ever providing any evidence that the graph is connected. Basically, we showed high vertex connectivity of the connected components of the flip graphs, and Lawson gave us the connectedness on top of it. Still, it might be interesting to see whether one can show, e.g., $(n-4)$-vertex connectivity for bistellar flip graphs of sets in convex and general position in $\mathbb {R}^3$. A first step is supplied: There are always at least $n-4$ flips for such sets [24, Proposition 3.6.19].

### Mixing Rate of the Random Flip Process

Last, but not least, a natural next question is to show expansion properties of the flip graphs, ideally yielding rapid mixing of the process of flipping random edges in the flip graph. This is known for points in convex positions, still with a big gap between the upper bound of $O(n^5)$ and the lower bound of $\Omega (n^{3/2})$ (McShine and Tetali 1998 [28], and Molloy et al. 2001 [30]). There are related results for lattice point sets by Caputo et al. 2013 [8].

## Footnotes

1 Links are a basic notion in the theory of simplicial and polytopal complexes. In terms of the flip complex $\mathbb {X}$ discussed above, *T* corresponds to a vertex of $\mathbb {X}$, and what we call link here is the 1-skeleton of the link (in the topological sense) of the vertex *T* in $\mathbb {X}$.

2 Since ${{\,\mathrm{\textsf {inc}\mathrm }\,}}{U} \le 1$, this also shows that if *U* induces a tree in *S*, then all points in *U* have degree exactly $3$.

3 Personal communication (2019).
